# Temporal Profile of Brain Gene Expression After Prey Catching Conditioning in an Anuran Amphibian

**DOI:** 10.3389/fnins.2019.01407

**Published:** 2020-01-14

**Authors:** Vern Lewis, Frédéric Laberge, Andreas Heyland

**Affiliations:** Integrative Biology, University of Guelph, Guelph, ON, Canada

**Keywords:** transcriptome, learning and memory, RNA sequencing, *Bombina orientalis*, neuronal plasticity, immediate-early genes

## Abstract

A key goal in modern neurobiology is to understand the mechanisms underlying learning and memory. To that end, it is essential to identify the patterns of gene expression and the temporal sequence of molecular events associated with learning and memory processes. It is also important to ascertain if and how these molecular events vary between organisms. In vertebrates, learning and memory processes are characterized by distinct phases of molecular activity involving gene transcription, structural change, and long-term maintenance of such structural change in the nervous system. Utilizing next generation sequencing techniques, we profiled the temporal expression patterns of genes in the brain of the fire-bellied toad *Bombina orientalis* after prey catching conditioning. The fire-bellied toad is a basal tetrapod whose neural architecture and molecular pathways may help us understand the ancestral state of learning and memory mechanisms in tetrapods. Differential gene expression following conditioning revealed activity in molecular pathways related to immediate early genes (IEG), cytoskeletal modification, axon guidance activity, and apoptotic processes. Conditioning induced early IEG activity coinciding with transcriptional activity and neuron structural modification, followed by axon guidance and cell adhesion activity, and late neuronal pruning. While some of these gene expression patterns are similar to those found in mammals submitted to conditioning, some interesting divergent expression profiles were seen, and differential expression of some well-known learning-related mammalian genes is missing altogether. These results highlight the importance of using a comparative approach in the study of the mechanisms of leaning and memory and provide molecular resources for a novel vertebrate model in the relatively poorly studied Amphibia.

## Introduction

Broadly defined, learning is the change in behavior that comes as a result of experience (Mackintosh, 1974; Rudy, 2014). Information about prior experiences is established and stored as memories in brain neuronal networks and allows for the subsequent modification of behavior. At the level of the neuron, information encoding is mediated by changes in the efficacy of synaptic transmission (Castillo, 2012; Squire et al., 2012). The persistent increase in synaptic efficacy is termed long-term potentiation (LTP), while the weakening of such efficacy is termed long-term depression; both presynaptic and post-synaptic processes mediate these phenomena. Among the molecular mechanisms of long-term memory and learning, our knowledge of LTP is most advanced. LTP involves structural modification of the synapse as well as growth of new synaptic connections. While many mechanisms underlie LTP, these mechanisms share distinct temporal phases: *induction* is the initial event that triggers LTP, *expression* is an established change in synaptic transmission, and *maintenance* is the process of stabilizing the change over time (Lamprecht, 2014; Schaefer et al., 2017). *Induction* of long-term memory formation is independent of protein synthesis and mediated by the depolymerization and lengthening of actin cytoskeletal elements in axons and dendrites (Lynch et al., 2007; Rudy, 2015a). *Induction* also involves activity-dependent Ca^+2^ second messenger signaling cascades which lead to the recruitment of AMPA receptors to the post-synaptic density, as well as activation of the MAPK-CREB pathway which results in learning-related gene transcription (Kandel, 2012). These early memory processes begin the events leading to long-term changes in the strength of synaptic transmission. Learning-related changes in neuroarchitecture also involve modification of larger neuronal structures by neurite growth or pruning, and possibly the birth and differentiation of new neurons in brain regions that display adult neurogenesis (Cameron and Glover, 2015; Augusto-Oliveira et al., 2019). After *induction*, *expression*, and *maintenance* of the change in synaptic transmission are needed for the consolidation of long-term memory. *Expression* depends on the establishment of mechanisms that potentiate synaptic efficacy. For example, AMPA receptors recruited to the post-synaptic density during *induction* are phosphorylated, leading to increased conductance post-synaptically. Additionally, the availability of neurotransmitters is increased pre-synaptically by increasing the number of synaptic vesicles available (Abraham and Williams, 2003). *Maintenance* of long-term synaptic change is the last phase of LTP, and it involves protein synthesis. Waves of delayed expression of many genes relating to signal transduction (*camk2a)*, cytoskeletal organization (*arc)* and transcriptional regulation (*c-fos* and *egr-1*) have been implicated in the persistence of synaptic changes and memories (Katche et al., 2010; Lisman et al., 2012). Functionally, the *maintenance* phase involves stabilizing the increase in AMPA receptors at synapses, as well as structural stabilization of synaptic modifications, such as synaptic enlargement (Desmond and Levy, 1986; Stewart et al., 2000; Blitzer, 2005; Rudy, 2015b). While much is known about the molecular pathways underlying vertebrate LTP (Malenka and Bear, 2004), there is still much to be learned especially in terms of the mechanisms underlying maintenance of stored memory.

Invertebrates have long been popular models to investigate learning and memory mechanisms due to the simplicity and accessibility of their nervous systems and vast behavioral repertoires. Many arthropods and cephalopods are capable of complex forms of learning like contextual, spatial, and concept learning (Mather and Kuba, 2013; Perry et al., 2013; Byrne and Hawkins, 2015). Our understanding of the molecular correlates of learning and memory comes from a large body of literature that started with work on *Aplysia californica*, *Caenorhabditis elegans*, and *Drosophila melanogaster* (Kandel and Schwartz, 1982; Sokolowski, 2001; Kaletta and Hengartner, 2006). Many molecular mechanisms underlying long-term memory formation are conserved between invertebrates and mammals (for reviews see Bailey et al., 1996; Cayre et al., 2002; Kandel et al., 2014). Long-term facilitation, the invertebrate analog of LTP, engages many similar molecular signaling pathways as mammalian LTP, moreover, both invertebrates and mammals show synaptic potentiation and depression as parallel processes underling memory formation and maintenance. However, while invertebrates share some learning mechanisms with vertebrates, robust information about the transition from invertebrate to vertebrate learning mechanisms is lacking in the literature. Invertebrates are incredibly diverse and important differences in central nervous system organization could limit their usefulness as comparative models for vertebrate learning. Furthermore, molecular differences in invertebrate and vertebrate learning mechanisms have not been studied in detail, and while some fundamental similarities exist, important differences have been noted, such as the absence of retrograde signaling pathways in invertebrates and different mechanisms of modulation of synaptic plasticity between groups (Glanzman, 2010; Shomrat et al., 2015). Lastly, it has not been determined if similarities in learning mechanisms represent homology or homoplasy, thus compounding the limited usefulness of invertebrates as comparative models. Such differences may suggest evolutionary divergence in some learning and memory mechanisms. Hence, the study of the molecular mechanisms involved learning could benefit from a broader selection of animals, especially representatives of basal vertebrate lineages, which are understudied. A basal vertebrate comparative model would help us to determine if learning mechanisms have been conserved or lost during the evolution of the complex behaviors demonstrated by amniote vertebrates.

Anuran amphibians, while generally understudied, are capable of several forms of learning (e.g., Schmajuk et al., 1980; Daneri et al., 2007; Mitchell and McCormick, 2013; Ramsay et al., 2013; Liu et al., 2016; Miller et al., 2018). The fire-bellied toad *Bombina orientalis* is a member of the *Bombinatoridae*, a group which occupies a basal position in anuran phylogeny (Yu et al., 2007) and follows a typical anuran life history with aquatic larvae and terrestrial adults, unlike the more established anuran models of the *Xenopus* genus which are derived aquatic specialists. *B. orientalis* offers a closer comparison with amniotes than phylogenetically more distant models, such as invertebrates or teleost fishes and shows extensive homology in brain structure with mammals (Laberge and Roth, 2007). Their phylogenetic position as a basal tetrapod, and brain homology, may help establish the ancestral tetrapod condition for brain structure and behavioral capacity, allowing us to address questions about the evolution of learning and memory.

Here we investigate the temporal sequence of gene expression after conditioning in the brain of the fire-bellied toad. We used a previously developed prey-catching conditioning task to initiate gene expression and assessed whole-brain gene expression by transcriptomic analysis at different time points following conditioning. The initial steps of short-term memory formation occur immediately after a learning event, and as such, transcriptomic analysis is not amenable to such short-term changes mediating early plasticity. Given that immediate early gene (IEG) expression is consistently associated with learning in vertebrates (Jarvis et al., 1995; Guzowski et al., 2001; Mokin and Keifer, 2005; Lau et al., 2011), and is activated very quickly and transiently, time-points must be considered which capture both IEG expression as well as related downstream genes whose expression depends on IEGs. Many IEGs are known to show expression as early as 30 min following a learning event and can persist for up to 4–6 h. Furthermore, there is evidence that structural changes of the neuron are still taking place for long periods after a learning event in mammals and late phase IEG activity appears to be a critical component for long-term memory persistence, for instance, *c-fos* shows late expression linked to the persistence of fear memory (Bekinschtein et al., 2008; Katche et al., 2010; Hertler et al., 2016). Therefore, early (2 h), intermediate (4 h), and late timepoints were included (24 h) in our study. If the underlying molecular pathways are conserved from anurans to mammals, we expect to see similar changes in transcription over time following conditioning. This study serves as a foundational step for investigating the molecular correlates of learning in *B. orientalis* and provides a new vertebrate model for the comparative study of the molecular mechanisms of learning and memory.

## Materials And Methods

### Animals

To quantify gene expression following conditioning, we trained 14 fire-bellied toads on a prey catching task in which the modification of a snapping response toward a visual prey stimulus by the administration of food reward was previously shown to involve learning (Ramsay et al., 2013). Animals were purchased from National Reptile Supply (Mississauga, ON) and housed in glass terrariums of 37 × 22 × 25 cm in dimension. Temperature was held constant at 21°C, and a 12/12 h light-dark photoperiod was maintained with light onset at 7 h. Terrariums were furnished with a rock-gravel substrate, broken clay pot pieces and large flat rocks for cover. The substrate was kept moist and the animals had constant access to water in a bowl. Prior to behavioral assays, toads were fed juvenile crickets (*Acheta domesticus*) dusted with vitamin and calcium supplements weekly, *ad libitum*. Individual toads were identified based on dorsal patterns of coloration. All procedures were approved by the University of Guelph animal care committee (animal utilization protocol #3590) under guidance from the Canadian Council on Animal Care.

### Testing Arena

**FIGURE 1:**
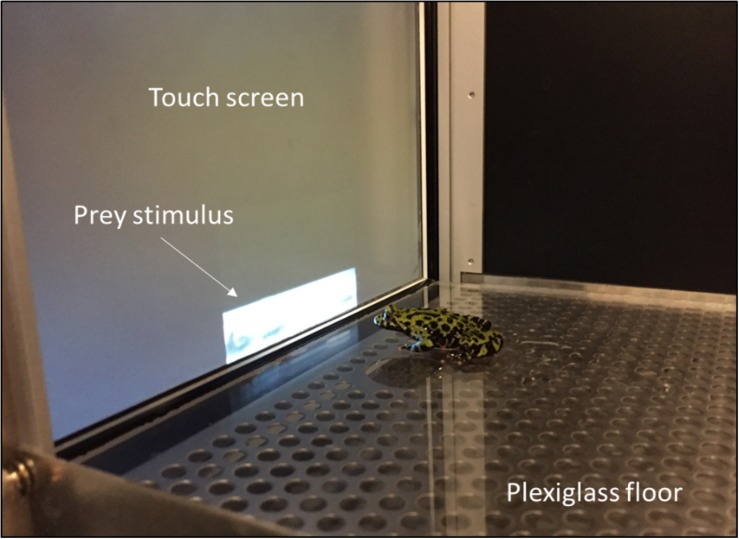
Training area for prey-catching conditioning. Toads were required to touch the prey stimulus five times, at which point the video was turned off and the toad fed a cricket (Photo by V. Lewis).

Behavioral assays were performed in a Bussey-Saksida rodent touch screen chamber running the ABET II software (Lafayette Instrument, Lafayette, IN, United States) (Figure 1). Each chamber wall and the touch screen were covered with matte vinyl coverings adhering to the interior surfaces to prevent the toads from reacting to their own reflections. At the end of every testing session, the Plexiglas floor and screen surfaces were cleaned with a 5% Tergazyme^TM^ solution and rinsed with distilled water to remove mucus build-up left by the toads. The visual stimulus used to stimulate toad prey catching was an 8 × 3 cm video of moving crickets against a white background previously used for similar conditioning experiments (Ramsay et al., 2013), located in the center-bottom portion of the monitor flush with the floor of the enclosure (Figure 1). The video was displayed using ABET VideoTouch version 2.18.10.2 (Lafayette Instruments, Lafayette, IN, United States).

### Conditioning

Prior to conditioning, each toad was gently handled for 1 min once per day over 5 days, followed by one shaping trial, and 2 days of rest. Shaping consisted of placing a toad in the touch screen chamber for 1 min, with hand feeding of one cricket after 30 s. No video stimulus was playing during shaping trials. All toads successfully completed shaping (consuming the cricket presented) on their first attempt. Two days after the shaping trial, toads underwent prey catching conditioning for a period of about 1.5 h. Conditioning involved two sessions of six trials. The trials within each session were separated by an interval of 3 min, while the two sessions were separated by 30 min. A preliminary experiment showed that this conditioning protocol was sufficient to see a significant change in performance between the two sessions (Supplementary Figure S2). In each trial, toads were required to snap five times at the cricket video stimulus before manual administration of a cricket reward by the experimenter. During each conditioning trial, a toad was placed in the arena facing the video stimulus from a distance of 15 cm and given 2 min to complete the task. The time it took toads to execute the five snaps (i.e., latency to task completion) was measured. Toads were placed temporarily in a separate container between trials and sessions. To insure high prey catching activity, toads were not fed for 7 days before shaping and the size of cricket rewards administered during conditioning was kept small to minimize satiation.

**FIGURE 2:**
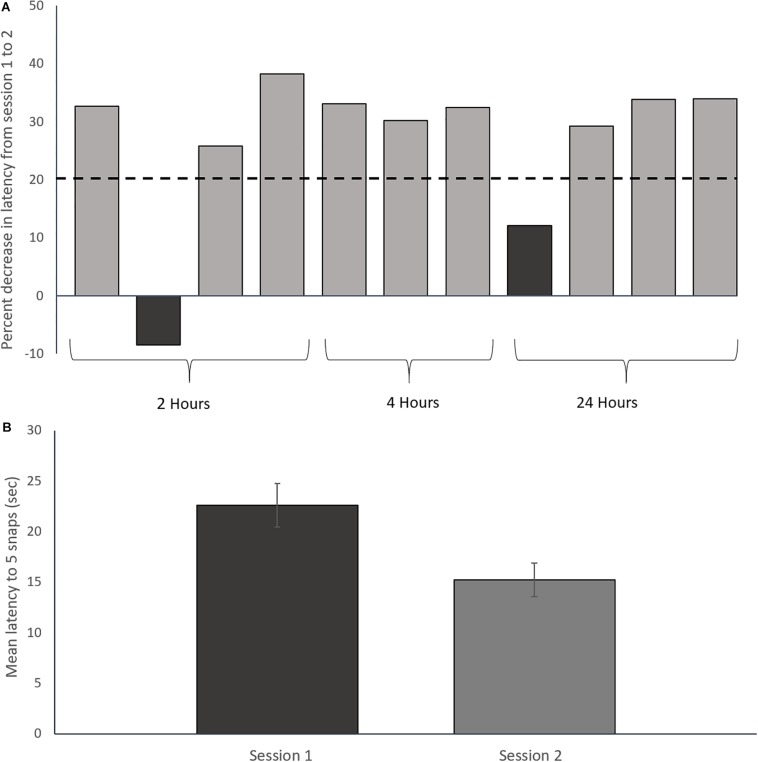
Prey catching conditioning results. **(A)** Percent decrease in latency to five snaps from session one to session two for each toad. The dashed line represents the rejection threshold, which was set to 1 standard deviation below the mean of two previous 2-session acquisition experiments (*n* = 20; 37% ± 15%; Lewis, unpublished). Dark bars are two rejected toads that were not used for transcriptomic analysis. Only toads showing a ≥20% decrease in latency between sessions 1 and 2 were used for transcriptomic analysis. **(B)** Mean prey-catching performance over 2 sessions of 6 trials for trained toads included in the study. Error bars represent ± Standard Deviation. The decrease in latency between sessions is statistically significant (Paired *t*-test: *t* = 9.07, *df* = 8, *p* < 0.01).

Following completion of the two conditioning sessions, only toads whose performance improved by a pre-defined amount were selected for genomic analysis. This performance criterion was based on the average change in performance (i.e., decreased latency to task completion) between sessions 1 and 2 (Figure 2A). Toads which showed less than 20% decrease in average latency between sessions were not used for genomic analysis. Two toads out of 14 were rejected based on this criterion.

### Time-Course

We chose to assess brain gene expression at 2, 4, and 24 h post-conditioning to evaluate gene expression associated with the induction, expression and maintenance phases of long-term memory. The temporal pattern of gene expression following conditioning was studied by sacrificing groups of toads (*n* = 3 per group) at the three time points post-training. Sacrifice timepoints were based on time elapsed from the start of the first conditioning trial in the first training session. Each toad was returned to its home terrarium during the interval between the end of conditioning and sacrifice. Control toads (*n* = 3) were housed with the conditioned toads, and handled daily, but never experienced the touch-screen chamber and were sacrificed immediately after being removed from the terrarium. We faced important constraints in our choice of controls. First, we could not use unpaired controls within the time period of 2 h before sampling of the first group of toads because Ramsay et al. (2013) showed that unpairing the prey catching response and cricket administration by 5 min was insufficient to prevent an improvement in prey caching performance with training. In fact, longer delays of 1 h and 1 day had to be used to demonstrate that learning was involved in modification of the innate prey catching response. Second, because prey catching toward a moving prey stimulus is an innate response, a control procedure where toads exposed to the cricket stimulus would not be rewarded could produce aversive learning and thus not be a good control. Third, similar to exposure to the cricket stimulus on its own, controls that would simply be put into the experimental context without the video stimulus playing and without cricket administration could also produce aversive learning because we have observed regularly that fire-bellied toads put into a restricted space try to escape if nothing is there to capture their attention (Laberge, personal observations). In the end, we chose to use unexposed controls as the best method to assess gene expression induced by the prey catching conditioning procedure. This method allows only the assessment of gene candidates that could be associated with learning. We verified the likelihood that the expression of these gene candidates was involved in learning by comparison with other studies (see section Discussion).

### Brain Dissection

Before sacrifice, toads were anesthetized by immersion in a 0.05% solution of buffered tricaine methanesulfonate (Argent Chemical Laboratories, Redmond, WA, United States) until loss of reaction to a leg pinch (about 5 min). Thereafter, the head was cut, and the brain was quickly dissected (∼10 min) from a ventral approach in a physiological Ringer’s solution consisting of Na^+^ 129 mM, K^+^ 4 mM, Ca ^2+^ 2.4 mM, Mg^2+^ 1.4 mM, Cl^–^ 115 mM, HCO^3–^ 25 mM, and glucose 10 mM. Cranial nerves, the pituitary gland and the dura mater were removed before transfer of the brain in 700 μl of TRIzol^TM^ (Life Technologies Inc., Carlsbad, CA, United States) followed by freezing on dry ice. The whole brain was used from the olfactory bulbs to the caudal hindbrain, excluding the spinal cord. Therefore, the pathways responsible for control of prey catching behavior (e.g., midbrain: Ewert, 1967; medulla: Takei et al., 1987; Matesz et al., 2014) and their potential modification by learning or other processes (e.g., Carr et al., 2002) were all included in the analysis of molecular responses.

### RNA Extraction and Sequencing

To isolate brain tissue RNA, brains were thawed and ground up using a pestle in a 1.5 ml Eppendorf tube before a modified TRIzol^TM^ extraction protocol was used (Supplementary Material, Section 2). Extracted RNA was cleaned up using the RNeasy mini kit (Qiagen, Venlo, Netherlands) before samples were stored at −80^*o*^C. RNA concentration and quality were respectively assessed using the NanoDrop1500 (Thermo Fisher Scientific) and the Agilent 2100 Bioanalyzer (Agilent Technologies; RIN ≥ 1.9; Supplementary Figure S4 and Supplementary Table S8).

Library preparation and sequencing was done by The Hospital for Sick Children’s Center for Applied Genomics (Toronto, ON, Canada). Libraries were poly-a filtered and 125 bp strand specific, paired-end sequenced reads were produced at a depth of 25 million reads with a HiSeq2500 system (Illumina Inc., CA, United States). All raw RNA-seq data has been uploaded to the Gene Expression Omnibus (GSE135054).

### Analysis Pipeline

#### Quality Control

Removal of indexing base pairs and trimming of low-quality segments was performed using the FastQC/Trimmomatic tools (Babraham Bioinformatics, Cambridgeshire, United Kingdom). Approximately 250 million reads across the 12 samples were trimmed for quality and length resulting in ∼230 million clean reads (Supplementary Table S1).

#### *De novo* Assembly- Alignment -Annotation

*De novo* assembly of the *B. orientalis* brain transcriptome was performed using custom scripts within the Trinity assembly suite, which reconstructs transcripts by partitioning RNA-seq data into multiple De Bruijn graphs (Haas et al., 2013). Read alignment was performed using the Bowtie2 − > eXpress pipeline, and differential gene expression was analyzed using DESeq2 (Langmead and Salzberg, 2012; Roberts and Pachter, 2013; Love et al., 2014). Transcript abundance was estimated in a genome-free manner using the alignment-based eXpress tool. eXpress aligns raw reads back against the *de novo* transcriptome and outputs expression-count estimates in Transcripts Per Million (TPM). DESeq2 uses these count estimates to analyze differential gene expression. Analysis was done in a pair-wise fashion, with each post-training time point being compared against the control. Differentially expressed genes (DEG) were sorted by a strict ≥ 1 log^2^ fold-change and the P-values reported by DESeq2 were used to calculate False Discovery Rates (FDR) for all transcripts using the Benjamini-Hochberg transformation (0.1 ≥ FDR, Benjamini and Hochberg, 1995). Annotation of the *de novo* transcriptome was performed using the Trinotate annotation suite (Haas et al., 2013), which is part of the Trinity platform. Trinotate takes the *de novo* assembly, uses the program Transdecoder (Haas et al., 2013) to estimate the longest open reading frames, and performs multiple BLAST homology searches against local databases (NCBI_nr, Swissprot, KEGG, GO).

Analysis of possible taxonomically-restricted anuran gene orthologs in the *B. orientalis* transcriptome was accomplished by homology search against model *Xenopus* spp. genomes. Two current *Xenopus* genomes (*X. laevis*, v2; *X. tropicalis*, v9.1) were combined to create a local database and a homology search was performed with the *B. orientalis* transcriptome using NCBI’s BLASTx tool (Altschul et al., 1990; Camacho et al., 2009). The resulting orthologs were then subjected to a protein motif survey to determine coding vs. non-coding transcripts. NCBI’s ORFinder website was used to find all open reading frames (ORF), then a motif search was performed for every ORF with an amino acid length greater or equal to 100. The motif survey was performed using the MyHits online portal (Pagni et al., 2007).

#### Hierarchical Clustering and Enrichment Analyses

**FIGURE 3:**
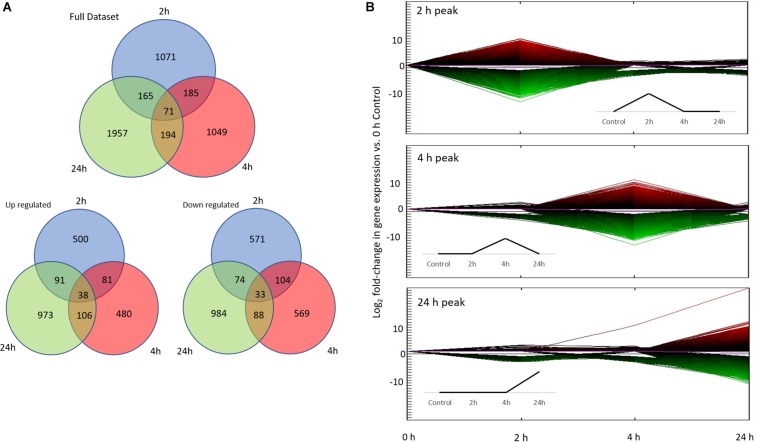
Differential gene expression associated with conditioning. **(A)** Number of differentially expressed genes (DEG) in each treatment (2, 4, 24 h) vs. control (0 h; total of 4692 DEG). The first Venn diagram includes the full data set, while the bottom two diagrams show up/down regulated gene numbers separately. **(B)** The top three co-expression patterns of all 122981 unsorted DEG based on hierarchical clustering. More than 90% of DEG fall into these three co-expression patterns: 2, 4, and 24 h peak. Line color represents direction and magnitude of gene expression; red is up regulated while green is down regulated. Inset figures are idealized up-regulated co-expression patterns.

Hierarchical clustering of co-expressed genes was performed using the Java-based desktop application Multi-Experiment Viewer (MeV; Chu et al., 2008) using K-means clustering with test parameters set to default (Pearson Correlation distance measure, Absolute distance used, and a Maximum Iteration of 50). Unsorted DEG in the dataset (122981 transcripts; Gene Omnibus: GSE135054) were sorted into 11 co-expression clusters. Gene Ontology enrichment analysis was performed on three clusters (2, 4, and 24 h absolute expression peaks; Figure 3) using the DAVID Bioinformatics Resources website and the PANTHER classification system (Thomas, 2003; Huang et al., 2009), with each cluster individually compared to a background gene list consisting of all gene annotations (*n* = 19,290 non-redundant annotated transcripts). The ratio of functional group genes to all genes in the set was compared between the individual clusters and the background, and functional groups were considered enriched if the number of genes in a cluster was significantly different compared to the background (Fisher’s exact test *p* ≥ 0.05). Finally, while Gene Ontology enrichment analysis provides an estimate of what functions may be affected by the treatment, they tell us little about possible molecular pathways that may be involved. In order to investigate the pathways involved, we searched the Kyoto Encyclopedia of Genes and Genomes (KEGG) database using the DAVID Bioinformatics resource and performed a similar enrichment analysis.

## Results

### Behavior

The 12 toads included in the study significantly improved prey catching performance between the first and second training sessions as demonstrated by a reduction in mean latency to complete 5 snaps between the training sessions (Figure 2B; paired *t*-test: *t* = 9.07, *df* = 8, *p* < 0.01; Supplementary Figure S3).

### Transcriptome Details

RNA-sequencing resulted in 268,925,312 raw reads across the 12 samples and yielded a total of 259,530,365 clean reads (96.5% of raw reads) following quality control procedures (Supplementary Table S1; Gene Omnibus: GSE135054). Transcriptome assembly resulted in 1,236,332 total transcripts consisting of 656,704,220 BP and ultimately 714,091 non-redundant “Trinity” genes (non-isoform transcripts; Supplementary Table S2). Replicate variability was tested using a correlation matrix comparing TPM values across treatments and clearly showed greater between- than within-treatment variability (Supplementary Figure S1). Alignment of raw reads back to the transcriptome resulted in an average of 23% uniquely mapped reads, with an average of 93% overall alignment quality across all samples (Supplementary Table S2).

As there is currently no publicly available genome for *B. orientalis*, 714,091 aligned transcripts were annotated using sequence Blast homology searches against the NCBI_nr and Swissprot protein databases, resulting in 137,305 transcripts annotated to 24,830 non-redundant genes (19.2% of transcriptome). All BLAST searches reported here were based on a cut-off of *E* ≥ 0.005 with a minimum Percent Similarity of 30%.

Differential expression was analyzed across three time points by comparing gene expression against the controls. A total of 122,981 contigs were found to be differentially expressed before sorting for fold change and FDR. Ultimately, a total of 4,692 transcripts were found to be significantly differentially expressed overall (*p* ≤ 0.1 FDR, | Fold change| ≥ 2; Figure 3). Of these, 1,344 annotated DEG were of use (557 genes with *Uncharacterized Protein* accessions were ignored for subsequent enrichment analysis). All subsequent expression profile and enrichment analyses were performed on these 1,344 annotated DEG.

Finally, a homology search of the 557 uncharacterized DEG against the *Xenopus* spp. database found 39 orthologs. Of these 39, 22 orthologs were upregulated and 17 were downregulated, with the majority (34) showing expression at a single timepoint (2, 4, or 24 h; Supplementary Table S6). Finally, 23 out of the 39 uncharacterized orthologs contained a protein motif, 15 of which showed a strong motif match (Sigrist, 2002), suggesting that most of these taxonomically-restricted transcripts were effectively coding for proteins (Supplementary Table S6).

### Identification of Distinct Temporal Co-expression Patterns

To analyze gene expression patterns over time (both up and down regulation of genes), we adapted the time points of our experiment based on *a priori* expectations informed by the literature on molecular responses associated with learning. These time points are: *Early* (2 h), *Intermediate* (4 h), *Late* (24 h) in comparison to the beginning of the experiment (0 h). We then performed an unbiased clustering analysis on the entire unsorted DEG dataset (122981 DEG), which resulted in 11 co-expression patterns based on absolute expression levels. Of the 11 co-expression patterns, the majority of DEG (>90%) were found in the *Early, Intermediate*, and *Late* co-expression peak profiles (Figure 3). Hence, all subsequent analyses were performed on these three co-expression profiles.

### Enrichment Analysis and Annotation of Co-expression Groups

**FIGURE 4:**
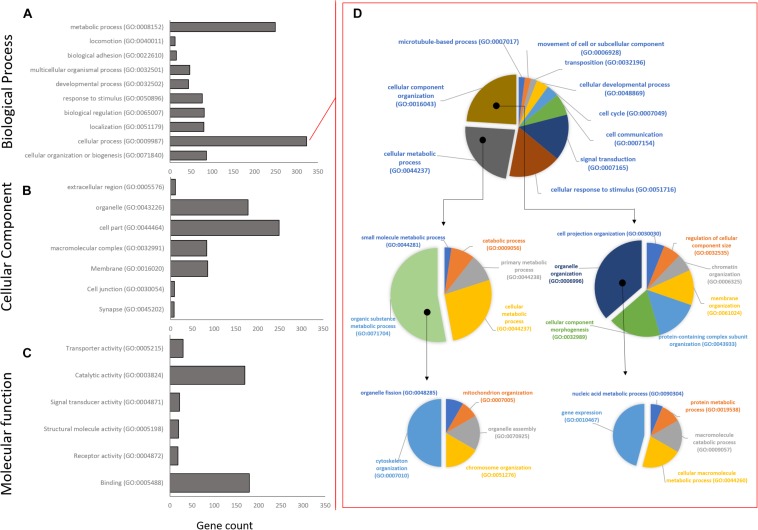
Distribution of Gene Ontology assignments for all annotated DEG transcripts. **(A)** Biological process functional group. **(B)** Cellular component group. **(C)** Molecular function group. Horizontal bars represent the number of DEG found in the associated level 1 Gene Ontology functional classification. **(D)** The largest Biological Process group, Cellular Process, has been expanded to show relative distribution of DEG at GO levels 2–4. Isolated segments represent expanded subgroup in subsequent charts on the right. The majority of DEG in the Biological Process group belong to cytoskeletal organization and gene expression.

High level gene ontology annotation (GO) was performed on the three co-expression profiles identified above. GO annotations are separated into three broad categories: biological processes (BP), cellular component (CC), and molecular function (MF). All genes were sorted into first level GO sub-groups (herein referred to as functional groups) within these categories (Figure 4). In the biological process category, 989 genes were assigned to eight functional groups with the largest number of genes within the cellular and metabolic processes groups (Figure 4A), indicating that at the highest GO functional group level most DEG are involved in processes related to cell metabolism, growth, and maintenance. The cellular process functional group was unpacked to give a better idea of the specific processes involved. Interestingly, most genes in this group fall into the cytoskeleton organization (GO:0007010), and gene expression (GO:0010467) functional groups by GO level 4 (Figure 4D). In the cellular component category, 620 genes were assigned to eight functional groups with cell part and organelles showing the greatest gene assignment (Figure 4B). Lastly, in the molecular function category, 441 genes were assigned to six functional groups, with catalytic activity and binding being most represented (Figure 4C).

**FIGURE 5:**
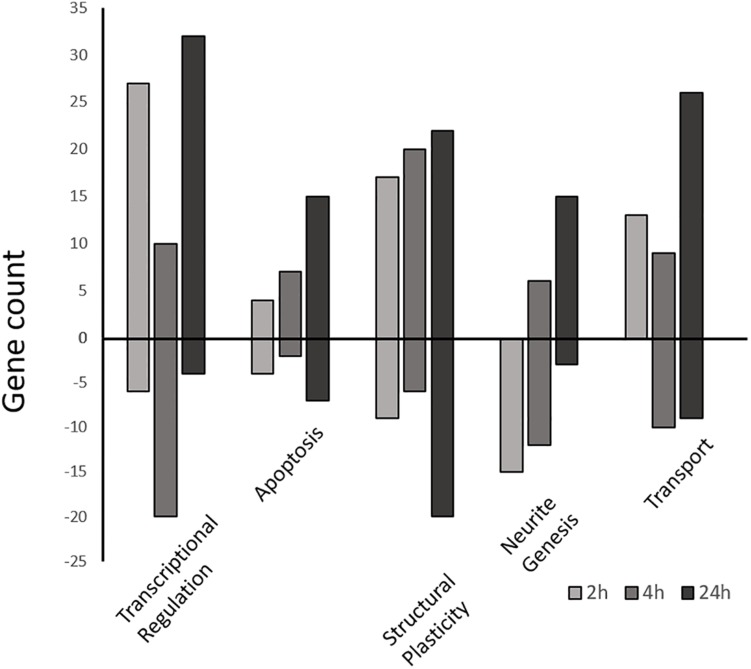
Differentially expressed gene counts associated with similar functional categories. Gene ontology enriched functional groups for each time-point (2, 4, and 24 h) were grouped by functional similarity and plotted as a function of gene number. Positive bars represent upregulated genes vs. the control, while negative bars are downregulated genes. All genes part of the enrichment analysis as well as manually selected genes (Supplementary Tables S4A–C) are included.

Following GO annotation, a functional group enrichment analysis was performed. Significant GO groups consisting of 2 or less genes were omitted. In total, the *Early* co-expression profile consisted of 10 BP, 16 CC, and 6 MF enriched functional groups, the *Intermediate* profile consisted of 18 BP, 8 CC, and 3 MF enriched groups, and the *Late* profile consisted of 11 BP, 7 CC, and 12 MF enriched groups (Figure 5 and Supplementary Tables S3A–C).

At the *Early* time point most upregulated genes are related to transcriptional regulation, followed by structural plasticity-related genes (actin filament and cytoskeleton organization; axon guidance; positive regulation of synapse assembly), and intracellular transport genes (transport, protein transport; Supplementary Table S3A). This activity is localized mainly to the nucleus, intracellularly, and to neuronal cell bodies, with some activity in the axon and growth cone. The MF group shows some minor protein dimerization activity but is dominated by DNA binding activity. Conversely, other groups related to structural plasticity and genesis of neuronal extensions are down-regulated at 2 h (neuron projection development, microtubule cytoskeleton organization).

The *Intermediate* time point shows up-regulation in several genes related to structural plasticity GO groups (axon guidance; single organismal cell-cell adhesion; homophilic cell adhesion via plasma membrane adhesion molecules; heterophilic cell-cell adhesion via plasma membrane cell adhesion molecules; cell-matrix adhesion; Figure 5 and Supplementary Table S3B) as well as endocytosis, apoptosis, and metabolic activity. The MF category shows nucleic acid binding activity, while activity localizes mainly to the mitochondrion, transcription factor complex, and filopodium. The most suppressed biological process group is regulation of transcription followed by protein modification, axonogenesis, and metabolic activity.

Finally, the *Late* time point also shows most up-regulated genes that are related to transcriptional regulation (regulation of transcription, DNA-templated; transcription, DNA-templated). Interestingly, while showing some structural plasticity gene enrichment (actin filament organization, focal adhesion assembly, cytoskeleton organization), and glucose metabolic gene enrichment (positive regulation of glucose import), the late profile shows strong up-regulation of apoptotic process genes (positive regulation of apoptotic process; Figure 5; Supplementary Table S3C). Conversely, the most suppressed groups include cell cycle, actin filament bundle assembly, and cell-cell adhesion. The *late* profile only shows molecular function activity in the Metabolic Processes and Binding groups. The binding group involves activity related to peptide binding (metal ion binding), DNA/RNA binding, and actin binding. While the metabolic group includes nuclease activity (endonuclease activity), proteolysis activity (aspartic-type endopeptidase activity) and polymerase activity (RNA-directed DNA polymerase activity). Furthermore, the most suppressed MF group is cadherin binding involved in cell-cell adhesion. Lastly, most enriched activity is localized to the spindle and nuclear matrix.

Enriched functional groups within the Biological Process category were used to group DEG under the following general, high level, subjectively selected functional headings: transcriptional regulation, structural plasticity, transport, apoptosis, and neurite genesis (Supplementary Tables S4A–C). In this way genes not represented in the enrichment analysis could be included and discussed. A total of 239 additional DEG from this study were ultimately included.

### Pathway Analysis

We searched the KEGG database using all 1,344 annotated DEG to identify putative biological pathways represented in our transcriptome (Table 1). Interestingly, pathways related to virus infection were enriched (hsa05169: Epstein-Barr, hsa05168: herpes simplex), as well as two structural plasticity pathways (hsa04360: Axon guidance, hsa04514: Cell adhesion molecules), and a protein processing pathway (hsa04141: Protein processing in endoplasmic reticulum).

**TABLE 1:** Enriched KEGG pathways, their constituent genes, and expression levels relative to control.

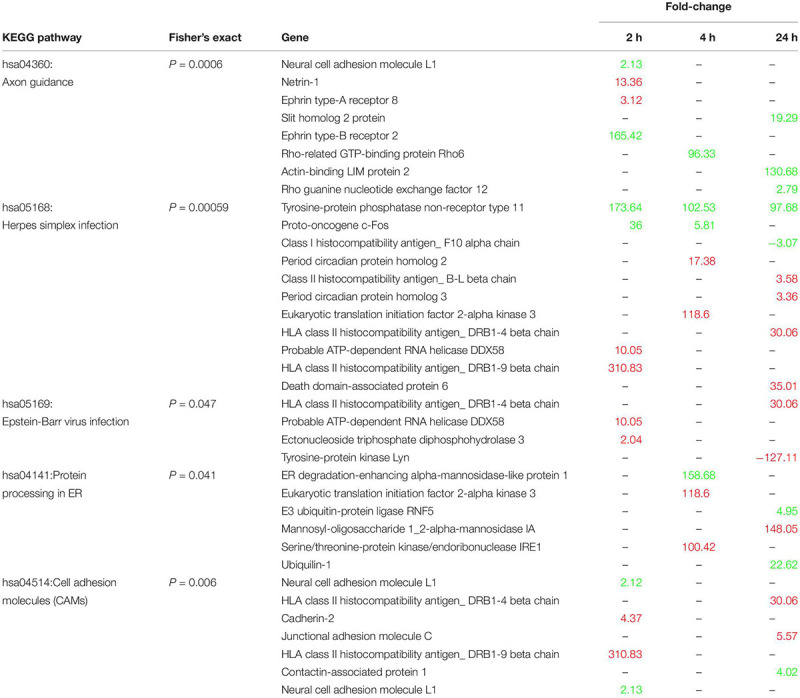

**Each gene within a given pathway is shown with its expression fold-change vs. the control.* Red is down-regulation and green is up-regulation.*

### Temporal Expression Patterns After Conditioning

There are currently over 100 described immediate early genes (IEG), and a subset of those are known to be induced during learning tasks (Clayton, 2000; Minatohara et al., 2016). Multiple learning related IEG’s were positively expressed at 2 h post-conditioning in *B. orientalis*, with a few at 4 h but none at 24 h (Table 2). Members of the Fos family *c-fos* and *fosB* were found to peak either early or intermediately and return to baseline by 24 h. The genes *arc/arg3.1, nr4a1*, and *egr1* were found to peak at 2 h, showed reduced expression by 4 h and returned to baseline by 24 h. The genes *jund* and *ier2* were both differentially expressed only in the 2 h treatment. Interestingly, *fosb* and *nr4a3*, which are known to be expressed early in response to acute stress or other environmental cues (30 min to 2 h; Perrotti et al., 2004; Hawk et al., 2012) were only differentially expressed at 4 h (73.5 and 21.1-fold-change) in our transcriptome. Further, *bdnf, homer1*, and *slc2a3*, which are known IEG’s associated with learning (Lu et al., 2008; Zhao et al., 2010; Chowdhury and Hell, 2018), were not differentially expressed even though they are present in the transcriptome.

**TABLE 2:** Expression levels (Fold-change relative to control) of the most relevant mammalian learning-associated genes at 2, 4, and 24 h.

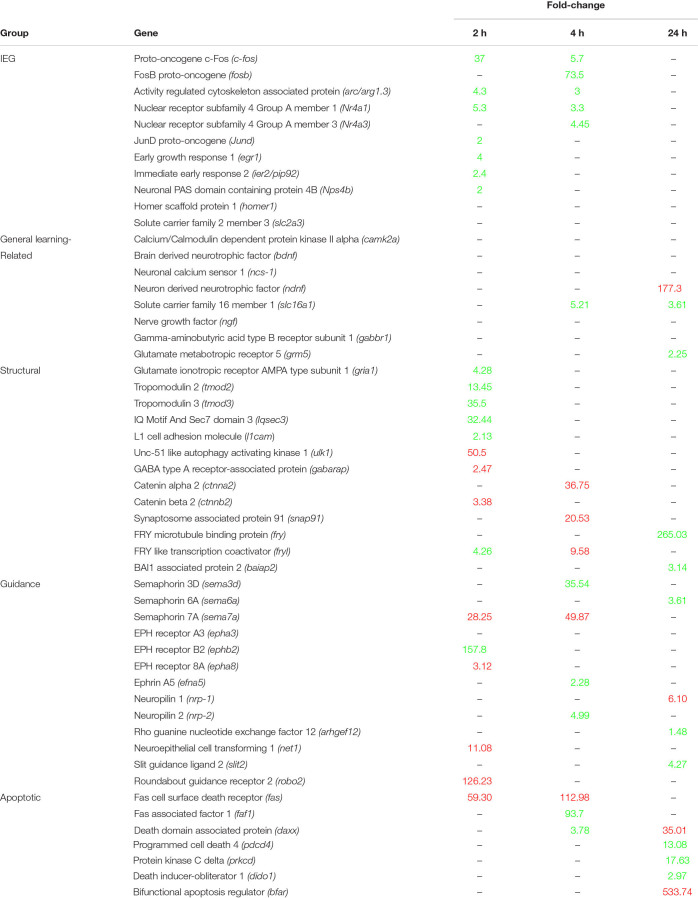

**The immediate-early gene (IEG) group represents any gene categorized as IEG. The General learning-related group represents genes chosen for their relevance to mammalian learning that do not fit into the other categories. The Structural group represents any gene involved in cytoskeletal modification. The Guidance group represents any gene involved in axon guidance. The Apoptotic group represents any gene involved in programmed cell death. Dashes (–) indicate no significant difference vs. the control. Green numbers are significantly upregulated while red numbers are downregulated. Where overlap in gene function exists, the gene was subjectively placed into the most relevant group.**

Based on the results of the enrichment and KEGG pathway analyses, the temporal expression of a number of important learning-related genes (based on the mammalian literature) was compared to mammalian expression patterns. These genes, including the aforementioned IEG’s, were grouped into generalized functional groups based roughly on gene ontology functional groups: *IEG’s*, *structural* genes, *axon guidance* genes, and *apoptotic* genes as well as a *general learning-related* category (Table 2). The *structural* group includes all genes related to cytoskeletal, microtubule, adhesion, actin, and tubulin activity, and consists of 13 DEG. *Structural* activity peaks at 2 h with 9 of 13 genes showing differential expression. *Axon guidance* includes any gene related to repulsion/attraction or extension of existing axons and consists of 11 DEG and 1 non-differentially expressed gene. *Axon guidance* activity is similar between time-points. *Apoptosis* included any genes associated with programmed cell death, neurite pruning and the intrinsic/extrinsic apoptotic pathways, and consists of 7 DEG, with activity peaking at 24 h for 5 of 7 genes showing differential expression. *General learning-related* is a catch all group for important mammalian learning genes which have only a few gene members (e.g., Signaling pathways, Neurotropic factors) or do not fit well into the other groups (e.g., *camk2a* or *ncs-1*) and consists of 14 DEG. Given that these genes were selected based on their importance in mammalian learning, it is interesting that most of them are not differentially expressed after prey catching conditioning in *B. orientalis*.

## Discussion

Prey-catching conditioning in *B. orientalis* engaged distinct temporal patterns of brain gene expression relating to structural and synaptic plasticity, programmed cell death, and transcriptional regulation; patterns which generally agree with functional patterns of gene expression after a learning event in other vertebrates (Bolhuis et al., 2000; Cavallaro et al., 2002; D’Agata and Cavallaro, 2003; Velho and Mello, 2008; Valles et al., 2011; Hertler et al., 2016). In *B. orientalis*, gene expression following conditioning starts with an increase in IEG expression at 2 h, which coincides with upregulation of gene expression associated with transcriptional regulation and structural plasticity (axon guidance, actin organization, synapse assembly). This is then followed at 4 h post-conditioning by an increase in axon guidance and cell adhesion activity, and a suppression of transcriptional regulation and axonogenesis. The last time point included in our experiment at 24 h is characterized by an increase in transcriptional regulation, apoptotic activity, and the formation of new neuronal projections. Functional group enrichment analysis highlighted structural plasticity, axon guidance, and programmed cell death as important changes in brain tissue induced by conditioning. While some similarities in the components of these pathways exist between mammals and the fire-bellied toad, and suggest phylogenetic conservation of learning pathways in tetrapods, many interesting differences are evident, specifically relating to the absence of well-known mammalian learning-related genes and the temporal patterns of axonogenesis.

### Immediate Early Genes

For the most part, the expression patterns of differentially expressed IEGs in response to prey-catching conditioning are consistent with previous literature on activity-dependent molecular responses in vertebrates (Pinaud, 2004; Burmeister and Fernald, 2005; Pinaud et al., 2006; Wada et al., 2006; Valles et al., 2011; Minatohara et al., 2016). The expression of *c-fos, egr-1, and acr/arg3.1* have been extensively studied and are consistently associated with neural stimulation and activity (Gallo et al., 2018). In rats peak *c-fos* expression generally ranges from 30 min to 2 h and returns to baseline around 4 h after a learning event (Cullinan et al., 1995; Barros et al., 2015). Similarly, *c-fos* peaks as soon as 30 min following song learning in zebra finches (Bolhuis et al., 2000). This resembles what we see in *B. orientalis*, with *c-fos* expression levels peaking early, and tapering to baseline by 24 h post-training. Similarly, *arc/arg3.1* and *nr4a1* expression levels, peak at 30–60 min, and lasts up to 4 h (*arc/arg3.1*: Plath et al., 2006; Bramham et al., 2010; *nr4a1*: Hawk et al., 2012). In *B. orientalis* these genes peak at 2 h, show reduced expression at 4 h and return to baseline by 24 h after training. Still, not all observed IEG expression patterns in *B. orientalis* are comparable to mammalian or bird patterns. The gene *fosb* is expected to show an early peak like *c-fos* (Nestler, 2015) but instead showed only high upregulation at 4 h in *B. orientalis*. *Homer1*, which is integral to the regulation of synaptic changes induced by LTP (Chowdhury and Hell, 2018), was not differentially expressed at any time-point after conditioning in *B. orientalis*, and neither was *bdnf*, a gene coding for an important neurotrophic factor associated with neurogenesis and long-term memory in mammals and song learning in birds (Lu et al., 2008; Wang et al., 2019). Therefore, despite observing important similarities in IEG expression between *B. orientalis* and other vertebrates, there were notable differences in that some IEGs showed different temporal patterns of expression while others were not engaged by the learning task used in the present study.

### Structural Modification

Gene ontology (GO) enrichment analysis highlighted several pathways which change as time progresses after prey catching conditioning, such as cytoskeletal modification, cell guidance, and cell death/genesis. We see gene expression early on (2–4 h) related to structural modification (actin cytoskeletal organization, axon guidance, adhesion, synapse assembly), then later expression of genes which mediate neurite genesis and apoptosis (Supplementary Table S4C). It appears that, like mammals, the establishment of new neural connections is started early following learning and transitions into neurite remodeling mechanisms later.

The early phase of memory formation in mammals involves actin cytoskeleton degradation and mobilization of glutamatergic receptors, followed by the consolidation of plasticity into long-term memory by the stabilization of these actin filaments (Abraham and Williams, 2003; Lynch et al., 2007). Interestingly, the early changes in our time course of gene expression revolve around structural modification and cytoskeletal organization, specifically in terms of actin organization. We see early expression of genes like the tropomodulins (*tmod2; tmod3*), which are involved in actin-capping and could help stabilize actin filaments, and the glutamate ionotropic receptor AMPA type subunit 1 (*gria1*), which is mobilized early in learning (Keifer and Zheng, 2010; Rao et al., 2014). Furthermore, several important genes involved in synaptic plasticity are upregulated early on: IQ motif and SEC7 domain-containing protein 3 (*iqsec3*), known to regulate inhibitory synapse formation (Um et al., 2016), neural cell adhesion molecule L1 (*l1cam*), involved in synaptic plasticity (Patzke et al., 2016), ephrin type-B receptor 2 (*ephb2*), involved in dendritic spine modification and excitatory synapse formation (Henkemeyer et al., 2003), and leucine-rich repeat-containing protein 24 (*lrrc24*), associated with synapse formation (Um et al., 2014). Since short-term memory formation is independent of protein synthesis (actin de/stabilization, AMPA receptor mobilization, etc.) and relies on cell stores of existing proteins, the gene expression observed at the earliest time point may represent turnover of these components.

Understanding the *late* expression of actin cytoskeletal genes observed in *B. orientalis*, as highlighted by GO enrichment analysis, is not straightforward. The structural genes expressed at 2–4 h are associated with *modification* of the neuron or neurites, while at 24 h the structural genes may be involved in neurite *genesis* or *destruction*. At 24 h there are several neuron projection development (GO:0031175) genes involved in neurite genesis or destruction that are differentially expressed. Interestingly, it appears that neurite modification is suppressed early following learning but is promoted as time progresses. At 2 h, genes like catenin beta-2 *(ctnnb2)*, serine/threonine-protein kinase ULK1 *(ulk1*), ephrin type-A receptor 8 *(epha8)*, and gamma-aminobutyric acid receptor-associated protein *(gabarap)*, associated with the generation of new neurites (Okazaki et al., 2000; Coyle et al., 2002; Gu et al., 2005; Lin et al., 2009), are highly suppressed. At 4 h the same pattern is observed, with suppression of genes like catenin alpha-2 (*ctnna2*; Schaffer et al., 2018), clathrin coat assembly protein AP180 (*snap91;* Bushlin et al., 2008), and protein furry homolog-like (*fryl*; Hayette et al., 2005). This contrasts with the late expression peak of neurite development genes like contactin-associated protein-like 2 (*cntnap2*; Poliak et al., 1999), brain-specific angiogenesis inhibitor 1-associated protein 2 (*baiap2*; Kang et al., 2016), and protein furry homolog (*fry*; Hayette et al., 2005) at 24 h. These neuron projection development (GO:0031175) genes are also cytoskeletal modification genes. Therefore, many of the GO highlighted late expressed cytoskeletal genes may be aiding in neurite genesis/destruction functions.

### Shared Molecular Pathways Involving Axon Guidance

We see gene expression associated with axon guidance activity at all timepoints, a well-known mechanism underlying learning and memory processes (Kolodkin and Tessier-Lavigne, 2011; Batool et al., 2019). The four main protein families involved in mammalian axon guidance are netrins, slit homolog proteins, ephrins, and semaphorins. Each family has representatives differentially expressed in our experiment.

The semaphorins 7a, 6a, and 3d (*sema7a/6a/3d*), which act to repulse growth cones or promote axon outgrowth, show differential expression across the treatments with *sema7a* downregulated at 2–4 h (outgrowth promoting; Jeroen Pasterkamp et al., 2003), *sema3d* upregulated at 4 h (axon repulsion; Wolman et al., 2004), and *sema6a* upregulated at 24 h (axon repulsion; Haklai-Topper et al., 2010). The semaphorins act by binding the receptor families plexins, neuropilins and integrins, however, expression of these receptors in *B. orientalis* is quite different from the expected mammalian pattern (Neufeld et al., 2002; Drabkin et al., 2014; Alto and Terman, 2017; Boyer and Gupton, 2018). We either saw a different receptor type expressed in toads, for example integrin alpha-7 (*itga7*) is downregulated at 24 h but it is not associated with semaphorin activity in mammals, or we saw no differential expression, as was the case with plexins. Further, while semaphorin 3d is known to bind neuropilin 1 (*nrp-1), nrp-1* was downregulated at 24 h in toads while the related *nrp-2* was upregulated along with *sema3d* at 4 h. Other growth cone manipulating genes differentially expressed in our experiment include the ligand netrin-1 (*net-1*), which is down-regulated at 2 h, and while its receptors are not differentially expressed, actin-binding LIM protein (*ablim*) is upregulated at 24 and is a downstream constituent of the *net-1* axon attraction pathway (Roof et al., 1997; Gitai et al., 2003). Lastly, the slit homolog 2 protein (*slit2*) ligand acts in axon repulsion and is highly upregulated at 24 h in toads, while its receptor roundabout homolog 2 (*robo2*) is downregulated at 2 h (Lopez-Bendito et al., 2007).

While axon repulsion seems to be a late stage event in conditioned *B. orientalis*, ephrin activity complicates this picture. The ephrin A5 ligand (*efna5*) is upregulated at 4 h and is known to bind to ephrin type-B receptor (*ephb2*; Himanen et al., 2004), which is highly upregulated at 2 h, while the ephrin type-A receptor 8 *(epha8*) is downregulated at 2 h. In mammals, Ephrin signaling was initially thought to be only repulsive but is now considered important for synaptic adhesion, and thus synaptogenesis (Lai and Ip, 2009). This is further evidenced by the fact that *ephb2* is known to produce an increase in NMDA receptors, enhancing LTP (Nolt et al., 2011). Ephrin activity in mammals has also been shown to be temporally mediated in that axon attraction and outgrowth is seen early, but eventually transitions into axon repulsion or withdrawal later (Liu et al., 2018).

While axon guidance pathways in conditioned *B. orientalis* appear to share some similarities with mammals, in terms of the genes involved and late axon repulsion, the overall patterns of expression are inconsistent. Axon outgrowth and attraction are early events during learning in mammals, but they appear to be late events in *B. orientalis*. Ultimately, axon guidance pathways appear to play an important role in tetrapods learning; however, the exact function of these pathways is not clear.

### Shared Molecular Pathways Involving Apoptosis

Functional group analysis showed enrichment of gene expression associated with apoptosis in the toad brains at 4 and 24 h post-conditioning. The genes involved are all part of the receptor-mediated “extrinsic” apoptosis pathway, which is well described in mammals (Elmore, 2007). Conversely, the “intrinsic” apoptosis pathway, which is based on the activation of mitochondrial pathways, does not seem to be part of learning-associated apoptotic processes. Interestingly, the apoptotic cysteine-aspartic proteases (caspases) and the B-cell lymphoma 2 (*bcl-2*) protein family, which are integral to apoptosis, are not differentially expressed across time points in conditioned toads. However, several genes in the apoptotic pathways show changes in expression.

The tumor necrosis factor receptor superfamily member 6 (*fas*) is a death receptor which mediates apoptosis through caspase 8 (Casp8) activation, or through the C-Jun N-terminal kinase (JNK) signaling pathway. The *fas* gene is strongly suppressed at 2–4 h post-conditioning in the toad brain, but FAS-associated factor 1 (*faf1*), which potentiates FAS-induced cell death (Chu et al., 1995), is highly upregulated at 4 h. Furthermore, the *fas* receptor-activated gene death domain-associated protein 6 (*daxx)*, which mediates JNK pathway activation and thus apoptosis, peaks at 4 h and is then suppressed at 24 h. The downstream effect of *fas-daxx-jnk* pathway activation is transcription of the *fas* receptor and its ligand (*fas-l;* Zhang J. et al., 2000). Perhaps, this suggests that while Fas/Faf1-mediated apoptotic activity begins at 4 h and lasts at least until 24 h, new *fas* receptors do not need to be mobilized until very late. Interestingly, programmed cell death protein 4 (*pdcd4*) and protein kinase C delta type (*prkcd)*, which peak at 24 h in the toad brain, are both associated with altered activity of the *fas-daxx-jnk* signaling pathway and the NF-kB signaling pathway, leading to pro-apoptotic activity (Emoto et al., 1995; Kilpatrick et al., 2002; Brodie and Blumberg, 2003; Lankat-Buttgereit and Göke, 2003). Finally, the apoptotic gene death-inducer obliterator 1 (*dido1*) is involved in the regulation of *pdcd4* and is also upregulated at 24 h post-training in the toad brain.

While we see evidence of activation of apoptotic pathways following conditioning in *B. orientalis*, the underlying purpose of this activation is unclear. One potential clue is that the apoptotic genes expressed in this study appear to modulate apoptotic pathways peripherally. Apoptosis is a well-known mechanism that shapes the developing nervous system, however, during adulthood, learning-related apoptosis activity may function as a pruning mechanism rather than a whole-cell death mechanism. This view is supported by findings showing that apoptosis mechanisms and neurite pruning share some molecular pathways (Riccomagno and Kolodkin, 2015; Mukherjee and Williams, 2017). For example, caspases 3 and 9 as well as the apoptosis regulator BAX (*bax*) are integral to axonal pruning (Simon et al., 2012; Cusack et al., 2013). Caspases are activated very quickly following induction of neurite pruning, but at a low level of expression, which prevents their full-blown apoptotic functions. While we did not see direct evidence of caspase or Bax/Bak activity in the toad brain, either the aforementioned *fas* apoptosis pathway or modulation of the Bax/Bak pathway could contribute to neurite pruning associated with learning. For example, the bifunctional apoptosis regulator (*bfar*) gene, which is suppressed at 24 h in toads, is known to interact with *bcl-2* and works to suppress the intrinsic *bax* activity (Zhang H. et al., 2000; Roth et al., 2003), which may suggest pro-pruning activity. Ultimately, while we see evidence that extrinsic apoptotic pathways similar to mammals are being activated, the results suggest that activation of these pathways in the late stages following prey catching conditioning underlie neuronal circuit refinement through neurite pruning rather than replacement of neurons through full blown cell death and birth of new neurons.

Interestingly, there is evidence that axon guidance gene families, such as the semaphorins and ephrins, also have neurite pruning functions. For example, *nrp-2* is known to form a complex with a plexin activated by the *sema3f* ligand to initiate pruning activity (Bagri et al., 2003; Schuldiner and Yaron, 2015). It is interesting then, that we see *nrp-2* upregulated with a semaphorin 3 family member at 4 h in this study. A reverse ephrin b3 (*efnb3)* signaling pathway (Eph − > Ephrin signaling, where the ephrin-B protein acts as a receptor; Xu and Henkemeyer, 2009), has also been implicated in neural pruning activity and while the *efnb3* is not differentially expressed in this study, it has been known to bind to the receptor *ephb2* (McClelland et al., 2010), which is upregulated at 2 h in the toad brain and may suggest pathway activation. Such early upregulation of *ephb2* may be a preparatory step for late pruning activity.

### Divergent Learning Pathways

Our study on a basal tetrapod provides further support for the hypothesis that basic learning pathways are conserved in animals. Several learning-related genes known from mammalian literature were expected to be differentially expressed in the late stages after conditioning in *B. orientalis*. However, while we saw differential expression of many of these genes, the expression patterns of some well-known learning associated genes were inconsistent with mammalian experiments. Genes like monocarboxylate transporter 1 (*slc16a1*; Takimoto and Hamada, 2014) and metabotropic glutamate receptor 5 *(grm5;* Jong et al., 2009) are differentially expressed at different times in toads in comparison to mammals, while calcium/calmodulin-dependent protein kinase 2A (*camk2a*), neuronal calcium sensor-1 (*ncs-1*), and nerve growth factor (*ngf*), which are all important learning-related genes in mammals (Weiss et al., 2010; Berry et al., 2012; Lisman et al., 2012), were not differentially expressed in this study. These inconsistencies could represent fundamental differences between the molecular mechanisms of learning in amphibians and other vertebrates. However, caution must be taken before concluding that such fundamental differences exist based on the present study. *B. orientalis* is a novel model in neurobiology and lacks a sequenced genome. Only 19% of expressed sequences and 30% of DEG were successfully annotated using our approach. This limited annotation likely had cascading effects on the pathway analyses presented here.

Poor genomic resources available for this species surely contributed to a low level of annotation, but Kwon (2015) also found thousands of taxonomically restricted genes in *Xenopus tropicalis* and *X. laevis* despite better genome resources. Interestingly, Kwon (2015) points out that *Xenopus laevis and X. tropicalis* genomes contains ∼18,000 taxonomically-restricted genes (Hellsten et al., 2010; Session et al., 2016), possibly suggesting an evolutionary divergence between amphibians and amniotes. Given the basal position of *B. orientalis*, there may be some divergence in the molecular mechanisms of learning compared to other more derived tetrapods; such divergence would be evidenced by the discovery of taxonomically-restricted anuran orthologs in *B. orientalis* learning-related gene expression. Indeed, a number of putative taxonomically-restricted gene orthologs were differentially expressed after conditioning in the *B. orientalis* transcriptome. Therefore, the presence of abundant taxonomically-restricted anuran genes could have contributed to the low annotation success of the *B. orientalis* brain tissue transcriptome. Additionally, differences in learning-related gene expression compared to other vertebrates may reflect divergent evolution of some learning and memory mechanisms in anuran amphibians.

### Methodological Limitations

In addition to poor annotation and the possibility of genomic divergence in vertebrates, the behavioral paradigm used to elicit gene expression may limit what molecular pathways are engaged. The many different tasks used to study the molecular correlates of learning in animals might not all engage comparable gene expression as the prey catching conditioning task used here. Furthermore, time point selection may not have adequately covered important periods of gene expression. While we are confident that the 2 h time point represented the peak of IEG expression based on preliminary qPCR data (Lewis, unpublished), the later time points were based on mammalian literature and light/dark cycle constraints. The 12 h time point was omitted from our experiment due to potential confounding effects of nighttime darkness on gene expression in the diurnal fire-bellied toad. Additionally, because conditioning procedures are difficult to implement in amphibians, our control method allowed only the assessment of candidate gene expression that could be associated with learning. Some gene expression could have instead been associated with exposure to the experimental context or prey stimulus independent of the association between prey catching and food reward. Thus, our conclusions about learning-related gene expression had to rely extensively on comparison with studies done in mammals because relevant gene expression studies are scarce in other animals. Despite the above constraints, our results highlight interesting avenues for future investigation and provide useful genomic resources for comparative research in the molecular basis of learning.

## Conclusion

This study represents the first in-depth transcriptional analysis of the temporal patterns of gene expression following conditioning in an amphibian. *Bombina orientalis* is a basal anuran which may provide clues about the ancestral state of learning and memory mechanisms in tetrapods and thus help evaluate the evolution of learning in vertebrates. Many similarities exist between the present results in *B. orientalis* and learning-related gene expression in mammals, especially with respect to IEG activity and structural plasticity of the neuron, but many interesting differences were also highlighted. It will be important to determine which of the gene candidates outlined in this study are directly associated with learning processes. This could be done by using a different conditioning task that is amenable to unpaired controls, although it could prove difficult to find such a task for work on amphibians. Alternatively, because extended prey catching training produces an increasingly inflexible and automatic response (Ramsay et al., 2013), these different phases of learning could be used to track gene expression as conditioning events progressively lose their potency to influence behavior. Further study of differences in molecular correlates of learning could provide important clues to help explain differences in cognition among vertebrates. Future work on the brain regions and circuits involved in conditioning in amphibians should also help with the latter.

## Data Availability Statement

The datasets generated for this study can be found in the NCBI gene omnibus; accession: GSE135054.

## Ethics Statement

The animal study was reviewed and approved by the University of Guelph animal care committee (animal utilization protocol #3590).

## Author Contributions

All authors listed have made a substantial, direct and intellectual contribution to the work, and approved it for publication.

## Conflict of Interest

The authors declare that the research was conducted in the absence of any commercial or financial relationships that could be construed as a potential conflict of interest.

## References

[B1] Abraham W. C. Williams J. M. (2003). Properties and mechanisms of LTP maintenance. *Neuroscientist* 9 463–474. 10.1177/1073858403259119 14678579

[B2] Alto L. T. Terman J. R. (2017). “Semaphorins and their signaling mechanisms,” in *Semaphorin Signaling*, ed. Terman J. R. (New York, NY: Springer), 1–25. 10.1007/978-1-4939-6448-2_1 PMC5538787 27787839

[B3] Altschul S. F. Gish W. Miller W. Myers E. W. Lipman D. J. (1990). Basic local alignment search tool. *J. Mol. Biol.* 215 403–410. 10.1006/jmbi.1990.9999 2231712

[B4] Augusto-Oliveira M. Arrifano G. Malva J. Crespo-Lopez M. (2019). Adult hiocampal neurogenesis in different taxonomic groups: possible functional similarities and striking controversies. *Cells* 8:125. 10.3390/cells8020125 30764477 PMC6406791

[B5] Bagri A. Cheng H.-J. Yaron A. Pleasure S. J. Tessier-Lavigne M. (2003). Stereotyped pruning of long hiocampal axon branches triggered by retraction inducers of the Semaphorin family. *Cell* 113 285–299. 10.1016/S0092-8674(03)00267-8 12732138

[B6] Bailey C. H. Bartsch D. Kandel E. R. (1996). Toward a molecular definition of long-term memory storage. *Proc. Natl. Acad. Sci. U.S.A.* 93 13445–13452. 10.1073/pnas.93.24.13445 8942955 PMC33629

[B7] Barros V. N. Mundim M. Galindo L. T. Bittencourt S. Porcionatto M. Mello L. E. (2015). The pattern of c-Fos expression and its refractory period in the brain of rats and monkeys. *Front. Cell. Neurosci.* 9:72. 10.3389/fncel.2015.00072 25814929 PMC4357304

[B8] Batool S. Raza H. Zaidi J. Riaz S. Hasan S. Syed N. I. (2019). Synapse formation: from cellular and molecular mechanisms to neurodevelopmental and neurodegenerative disorders. *J. Neurophysiol.* 121 1381–1397. 10.1152/jn.00833.2018 30759043

[B9] Bekinschtein P. Cammarota M. Katche C. Slipczuk L. Rossato J. I. Goldin A. (2008). BDNF is essential to promote persistence of long-term memory storage. *Proc. Natl. Acad. Sci. U.S.A.* 105 2711–2716. 10.1073/pnas.0711863105 18263738 PMC2268201

[B10] Benjamini Y. Hochberg Y. (1995). Controlling the false discovery rate: a practical and powerful aroach to multiple testing. *J. R. Stat. Soc. B* 57 289–300. 10.1111/j.2517-6161.1995.tb02031.x

[B11] Berry A. Bindocci E. Alleva E. (2012). NGF, brain and behavioral plasticity. *Neural Plast.* 2012 1–9. 10.1155/2012/784040 22474604 PMC3306960

[B12] Blitzer R. D. (2005). Long-term potentiation: mechanisms of induction and maintenance. *Sci. Signal.* 2005:tr26. 10.1126/stke.3092005tr26 16278490

[B13] Bolhuis J. J. Zijlstra G. G. O. den Boer-Visser A. M. Van der Zee E. A. (2000). Localized neuronal activation in the zebra finch brain is related to the strength of song learning. *Proc. Natl. Acad. Sci. U.S.A.* 97 2282–2285. 10.1073/pnas.030539097 10681421 PMC15792

[B14] Boyer N. P. Gupton S. L. (2018). Revisiting Netrin-1: one who guides (Axons). *Front. Cell. Neurosci.* 12:221. 10.3389/fncel.2018.00221 30108487 PMC6080411

[B15] Bramham C. R. Alme M. N. Bittins M. Kuipers S. D. Nair R. R. Pai B. (2010). The Arc of synaptic memory. *Exp. Brain Res.* 200 125–140. 10.1007/s00221-009-1959-2 19690847 PMC2803749

[B16] Brodie C. Blumberg P. M. (2003). Regulation of cell apoptosis by protein kinase c δ. *Apoptosis* 8 19–27. 12510148 10.1023/a:1021640817208

[B17] Burmeister S. S. Fernald R. D. (2005). Evolutionary conservation of the egr-1 immediate-early gene response in a teleost. *J. Comp. Neurol.* 481 220–232. 10.1002/cne.20380 15562507

[B18] Bushlin I. Petralia R. S. Wu F. Harel A. Mughal M. R. Mattson M. P. (2008). Clathrin assembly protein AP180 and CALM differentially control axogenesis and dendrite outgrowth in embryonic hiocampal neurons. *J. Neurosci.* 28 10257–10271. 10.1523/JNEUROSCI.2471-08.2008 18842885 PMC2581865

[B19] Byrne J. H. Hawkins R. D. (2015). Nonassociative learning in invertebrates. *Cold Spring Harb. Perspect. Biol.* 7:a021675. 10.1101/cshperspect.a021675 25722464 PMC4448621

[B20] Camacho C. Coulouris G. Avagyan V. Ma N. Papadopoulos J. Bealer K. (2009). BLAST+: architecture and alications. *BMC Bioinformatics* 10:421. 10.1186/1471-2105-10-421 20003500 PMC2803857

[B21] Cameron H. A. Glover L. R. (2015). Adult neurogenesis: beyond learning and memory. *Annu. Rev. Psychol.* 66 53–81. 10.1146/annurev-psych-010814-015006 25251485 PMC5612417

[B22] Carr J. A. Brown C. L. Mansouri R. Venkatesan S. (2002). Neuropeptides and amphibian prey-catching behavior. *Comp. Biochem. Physiol.* 132B 151–162. 10.1016/S1096-4959(01)00545-0 11997218

[B23] Castillo P. E. (2012). Presynaptic LTP and LTD of excitatory and inhibitory synapses. *Cold Spring Harb. Perspect. Biol.* 4:a005728. 10.1101/cshperspect.a005728 22147943 PMC3281573

[B24] Cavallaro S. D’Agata V. Manickam P. Dufour F. Alkon D. L. (2002). Memory-specific temporal profiles of gene expression in the hiocampus. *Proc. Natl. Acad. Sci. U.S.A.* 99 16279–16284. 10.1073/pnas.242597199 12461180 PMC138602

[B25] Cayre M. Malaterre J. Scotto-Lomassese S. Strambi C. Strambi A. (2002). The common properties of neurogenesis in the adult brain: from invertebrates to vertebrates. *Comp. Biochem. Physiol.* 132B 1–15. 10.1016/S1096-4959(01)00525-5 11997205

[B26] Chowdhury D. Hell J. W. (2018). Homeostatic synaptic scaling: molecular regulators of synaptic AMPA-type glutamate receptors. *F1000Research* 7:234. 10.12688/f1000research.13561.1 29560257 PMC5832907

[B27] Chu K. Niu X. Williams L. T. (1995). A Fas-associated protein factor, FAF1, potentiates Fas-mediated apoptosis. *Proc. Natl. Acad. Sci. U.S.A.* 92 11894–11898. 10.1073/pnas.92.25.11894 8524870 PMC40509

[B28] Chu V. T. Gottardo R. Raftery A. E. Bumgarner R. E. Yeung K. (2008). MeV+R: using MeV as a graphical user interface for bioconductor alications in microarray analysis. *Genome Biol.* 9:R118. 10.1186/gb-2008-9-7-r118 18652698 PMC2530872

[B29] Clayton D. F. (2000). The genomic action potential. *Neurobiol. Learn. Mem.* 74 185–216. 10.1006/nlme.2000.3967 11031127

[B30] Coyle J. E. Qamar S. Rajashankar K. R. Nikolov D. B. (2002). Structure of GABARAP in two conformations: implications for GABAA receptor localization and tubulin binding. *Neuron* 33 63–74. 10.1016/s0896-6273(01)00558-x 11779480

[B31] Cullinan W. E. Herman J. P. Battaglia D. F. Akil H. Watson S. J. (1995). Pattern and time course of immediate early gene expression in rat brain following acute stress. *Neuroscience* 64 477–505. 10.1016/0306-4522(94)00355-9 7700534

[B32] Cusack C. L. Swahari V. Hampton Henley W. Michael Ramsey J. Deshmukh M. (2013). Distinct pathways mediate axon degeneration during apoptosis and axon-specific pruning. *Nat. Commun.* 4:1876. 10.1038/ncomms2910 23695670 PMC4183061

[B33] D’Agata V. Cavallaro S. (2003). Hiocampal gene expression profiles in passive avoidance conditioning. *Eur. J. Neurosci.* 18 2835–2841. 10.1111/j.1460-9568.2003.03025.x 14656332

[B34] Daneri M. F. Papini M. R. Muzio R. N. (2007). Common toads (*Bufo arenarum*) learn to anticipate and avoid hypertonic saline solutions. *J. Comp. Psychol.* 121 419–427. 10.1037/0735-7036.121.4.419 18085926

[B35] Desmond N. L. Levy W. B. (1986). Changes in the postsynaptic density with long-term potentiation in the dentate gyrus. *J. Comp. Neurol.* 253 476–482. 10.1002/cne.902530405 3025273

[B36] Drabkin H. Nasarre P. Gemmill R. (2014). The emerging role of class-3 semaphorins and their neuropilin receptors in oncology. *OncoTargets Therapy* 7 1663–1687. 10.2147/OTT.S37744 25285016 PMC4181631

[B37] Elmore S. (2007). Apoptosis: a review of programmed cell death. *Toxicol. Pathol.* 35 495–516. 10.1080/01926230701320337 17562483 PMC2117903

[B38] Emoto Y. Manome Y. Meinhardt G. Kisaki H. Kharbanda S. Robertson M. (1995). Proteolytic activation of protein kinase C delta by an ICE-like protease in apoptotic cells. *EMBO J.* 14 6148–6156. 10.1002/j.1460-2075.1995.tb00305.x 8557034 PMC394739

[B39] Ewert J.-P. (1967). Aktivierung der Verhaltensfolge beim Beutefang der Erdkröte (*Bufo bufo* L.) durch elektrische Mittelhirn-Reizung. *Zeitshrift für Vergleichende Physiologie* 54 455–481. 10.1007/BF00298232

[B40] Gallo F. T. Katche C. Morici J. F. Medina J. H. Weisstaub N. V. (2018). Immediate early genes, memory and psychiatric disorders: focus on c-Fos, Egr1 and Arc. *Front. Behav. Neurosci.* 12:79. 10.3389/fnbeh.2018.00079 29755331 PMC5932360

[B41] Gitai Z. Yu T. W. Lundquist E. A. Tessier-Lavigne M. Bargmann C. I. (2003). The netrin receptor UNC-40/DCC stimulates axon attraction and outgrowth through enabled and, in parallel, Rac and UNC-115/AbLIM. *Neuron* 37 53–65. 10.1016/S0896-6273(02)01149-2 12526772

[B42] Glanzman D. L. (2010). Common mechanisms of synaptic plasticity in vertebrates and invertebrates. *Curr. Biol.* 20 R31–R36. 10.1016/j.cub.2009.10.023 20152143 PMC5125775

[B43] Gu C. Shim S. Shin J. Kim J. Park J. Han K. (2005). The EphA8 receptor induces sustained MAP kinase activation to promote neurite outgrowth in neuronal cells. *Oncogene* 24 4243–4256. 10.1038/sj.onc.1208584 15782114

[B44] Guzowski J. F. Setlow B. Wagner E. K. McGaugh J. L. (2001). Experience-dependent gene expression in the rat hiocampus after spatial learning: a comparison of the immediate-early genes *Arc*, c- *fos*, and *zif268*. *J. Neurosci.* 21 5089–5098. 10.1523/JNEUROSCI.21-14-05089.2001 11438584 PMC6762831

[B45] Haas B. J. Papanicolaou A. Yassour M. Grabherr M. Blood P. D. Bowden J. (2013). De novo transcript sequence reconstruction from RNA-seq using the Trinity platform for reference generation and analysis. *Nat. Protoc.* 8 1494–1512. 10.1038/nprot.2013.084 23845962 PMC3875132

[B46] Haklai-Toer L. Mlechkovich G. Savariego D. Gokhman I. Yaron A. (2010). Cis interaction between Semaphorin6A and Plexin-A4 modulates the repulsive response to Sema6A. *EMBO J.* 29 2635–2645. 10.1038/emboj.2010.147 20606624 PMC2928682

[B47] Hawk J. D. Bookout A. L. Poplawski S. G. Bridi M. Rao A. J. Sulewski M. E. (2012). NR4A nuclear receptors suort memory enhancement by histone deacetylase inhibitors. *J. Clin. Invest.* 122 3593–3602. 10.1172/JCI64145 22996661 PMC3461922

[B48] Hayette S. Cornillet-Lefebvre P. Tigaud I. Struski S. Forissier S. Berchet A. (2005). *AF4p12*, a human homologue to the *furry* Gene of *Drosophila*, as a novel *MLL* fusion partner. *Cancer Res.* 65 6521–6525. 10.1158/0008-5472.CAN-05-1325 16061630

[B49] Hellsten U. Harland R. M. Gilchrist M. J. Hendrix D. Jurka J. Kapitonov V. (2010). The genome of the western clawed frog *Xenopus tropicalis*. *Science* 328 633–636. 10.1126/science.1183670 20431018 PMC2994648

[B50] Henkemeyer M. Itkis O. S. Ngo M. Hickmott P. W. Ethell I. M. (2003). Multiple EphB receptor tyrosine kinases shape dendritic spines in the hiocampus. *J. Cell Biol.* 163 1313–1326. 10.1083/jcb.200306033 14691139 PMC1435730

[B51] Hertler B. Buitrago M. M. Luft A. R. Hosp J. A. (2016). Temporal course of gene expression during motor memory formation in primary motor cortex of rats. *Neurobiol. Learn. Mem.* 136 105–115. 10.1016/j.nlm.2016.09.018 27686277

[B52] Himanen J.-P. Chumley M. J. Lackmann M. Li C. Barton W. A. Jeffrey P. D. (2004). Repelling class discrimination: ephrin-A5 binds to and activates EphB2 receptor signaling. *Nat. Neurosci.* 7 501–509. 10.1038/nn1237 15107857

[B53] Huang D. W. Sherman B. T. Lempicki R. A. (2009). Systematic and integrative analysis of large gene lists using DAVID bioinformatics resources. *Nat. Protoc.* 4 44–57. 10.1038/nprot.2008.211 19131956

[B54] Jarvis E. D. Mello C. V. Nottebohm F. (1995). Associative learning and stimulus novelty influence the song-induced expression of an immediate early gene in the canary forebrain. *Learn. Mem.* 2 62–80. 10.1101/lm.2.2.62 10467567

[B55] Jeroen Pasterkamp R. Peschon J. J. Spriggs M. K. Kolodkin A. L. (2003). Semaphorin 7A promotes axon outgrowth through integrins and MAPKs. *Nature* 424 398–405. 10.1038/nature01790 12879062

[B56] Jong Y. J. I. Kumar V. O’Malley K. L. (2009). Intracellular metabotropic glutamate receptor 5 (mGluR5) activates signaling cascades distinct from cell surface counterparts. *J. Biol. Chem.* 284 35827–35838. 10.1074/jbc.M109.046276 19840937 PMC2791012

[B57] Kaletta T. Hengartner M. O. (2006). Finding function in novel targets: *C. elegans* as a model organism. *Nat. Rev. Drug Discov.* 5 387–399. 10.1038/nrd2031 16672925

[B58] Kandel E. R. (2012). The molecular biology of memory: cAMP, PKA, CRE, CREB-1, CREB-2, and CPEB. *Mol. Brain* 5:14. 10.1186/1756-6606-5-14 22583753 PMC3514210

[B59] Kandel E. R. Dudai Y. Mayford M. R. (2014). The molecular and systems biology of memory. *Cell* 157 163–186. 10.1016/j.cell.2014.03.001 24679534

[B60] Kandel E. R. Schwartz J. H. (1982). Molecular biology of an elementary form of learning: modulation of transmitter release by cAMP. *Science* 218 433–443. 10.1126/science.6289442 6289442

[B61] Kang J. Park H. Kim E. (2016). IRSp53/BAIAP2 in dendritic spine development, NMDA receptor regulation, and psychiatric disorders. *Neuropharmacology* 100 27–39. 10.1016/j.neuropharm.2015.06.019 26275848

[B62] Katche C. Bekinschtein P. Slipczuk L. Goldin A. Izquierdo I. A. Cammarota M. (2010). Delayed wave of c-Fos expression in the dorsal hiocampus involved specifically in persistence of long-term memory storage. *Proc. Natl. Acad. Sci. U.S.A.* 107 349–354. 10.1073/pnas.0912931107 20018662 PMC2806699

[B63] Keifer J. Zheng Z. (2010). AMPA receptor trafficking and learning: AMPAR trafficking and learning. *Eur. J. Neurosci.* 32 269–277. 10.1111/j.1460-9568.2010.07339.x 20646058 PMC3985283

[B64] Kilpatrick L. E. Lee J. Y. Haines K. M. Campbell D. E. Sullivan K. E. Korchak H. M. (2002). A role for PKC-δ and PI 3-kinase in TNF-α-mediated antiapoptotic signaling in the human neutrophil. *Am. J. Physiol. Cell Physiol.* 283 C48–C57. 10.1152/ajpcell.00385.2001 12055072

[B65] Kolodkin A. L. Tessier-Lavigne M. (2011). Mechanisms and molecules of neuronal wiring: a primer. *Cold Spring Harb. Perspect. Biol.* 3:a001727. 10.1101/cshperspect.a001727 21123392 PMC3098670

[B66] Kwon T. (2015). Benchmarking transcriptome quantification methods for duplicated genes in *Xenopus laevis*. *Cytogenet. Genome Res.* 145 253–264. 10.1159/000431386 26112470

[B67] Laberge F. Roth G. (2007). Organization of the sensory input to the telencephalon in the fire-bellied toad, *Bombina orientalis*. *J. Comp. Neurol.* 502 55–74. 10.1002/cne.21297 17335050

[B68] Lai K. O. Ip N. Y. (2009). Synapse development and plasticity: roles of ephrin/Eph receptor signaling. *Curr. Opin. Neurobiol.* 19 275–283. 10.1016/j.conb.2009.04.009 19497733

[B69] Lamprecht R. (2014). The actin cytoskeleton in memory formation. *Prog. Neurobiol.* 117 1–19. 10.1016/j.pneurobio.2014.02.001 24530292

[B70] Langmead B. Salzberg S. L. (2012). Fast gaed-read alignment with Bowtie 2. *Nat. Methods* 9 357–359. 10.1038/nmeth.1923 22388286 PMC3322381

[B71] Lankat-Buttgereit B. Göke R. (2003). Programmed cell death protein 4 (pdcd4): a novel target for antineoplastic therapy? *Biol. Cell* 95 515–519. 10.1016/j.biolcel.2003.09.003 14630388

[B72] Lau B. Y. Mathur P. Gould G. G. Guo S. (2011). Identification of a brain center whose activity discriminates a choice behavior in zebrafish. *Proc. Natl. Acad. Sci. U.S.A.* 108 2581–2586. 10.1073/pnas.1018275108 21262817 PMC3038752

[B73] Lin C. C. Chou C. H. Howng S. L. Hsu C. Y. Hwang C. C. Wang C. (2009). GSKIP, an inhibitor of GSK3Î2, mediates the N-cadherin/Î2-catenin pool in the differentiation of SH-SY5Y cells. *J. Cell. Biochem.* 108 1325–1336. 10.1002/jcb.22362 19830702

[B74] Lisman J. Yasuda R. Raghavachari S. (2012). Mechanisms of CaMKII action in long-term potentiation. *Nat. Rev. Neurosci.* 13 169–182. 10.1038/nrn3192 22334212 PMC4050655

[B75] Liu X. D. Zhu X. N. Halford M. M. Xu T. L. Henkemeyer M. Xu N. J. (2018). Retrograde regulation of mossy fiber axon targeting and terminal maturation via postsynaptic Lnx1. *J. Cell Biol.* 217 4007–4024. 10.1083/jcb.201803105 30185604 PMC6219728

[B76] Liu Y. Day L. B. Summers K. Burmeister S. S. (2016). Learning to learn: advanced behavioural flexibility in a poison frog. *Anim. Behav.* 111 167–172. 10.1016/j.anbehav.2015.10.018

[B77] Lopez-Bendito G. Flames N. Ma L. Fouquet C. Di Meglio T. Chedotal A. (2007). Robo1 and Robo2 cooperate to control the guidance of major axonal tracts in the mammalian forebrain. *J. Neurosci.* 27 3395–3407. 10.1523/JNEUROSCI.4605-06.2007 17392456 PMC6672128

[B78] Love M. I. Huber W. Anders S. (2014). Moderated estimation of fold change and dispersion for RNA-seq data with DESeq2. *Genome Biol.* 15:550. 10.1186/s13059-014-0550-8 25516281 PMC4302049

[B79] Lu Y. Christian K. Lu B. (2008). BDNF: a key regulator for protein synthesis-dependent LTP and long-term memory? *Neurobiol. Learn. Mem.* 89 312–323. 10.1016/j.nlm.2007.08.018 17942328 PMC2387254

[B80] Lynch G. Rex C. S. Gall C. M. (2007). LTP consolidation: substrates, explanatory power, and functional significance. *Neuropharmacology* 52 12–23. 10.1016/j.neuropharm.2006.07.027 16949110

[B81] Mackintosh N. J. (1974). *The Psychology of Animal Learning.* Oxford: Academic Press.

[B82] Malenka R. C. Bear M. F. (2004). LTP and LTD: an embarrassment of riches. *Neuron* 44 5–21. 15450156 10.1016/j.neuron.2004.09.012

[B83] Matesz K. Kecskes S. Bácskai T. Rácz É Birinyi A. (2014). Brainstem circuits underlying the prey-catching behavior of the frog. *Brain Behav. Evol.* 83 104–111. 10.1159/000357751 24776991

[B84] Mather J. A. Kuba M. J. (2013). The cephalopod specialties: complex nervous system, learning, and cognition. *Can. J. Zool.* 91 431–449. 10.1139/cjz-2013-0009

[B85] McClelland A. C. Hruska M. Coenen A. J. Henkemeyer M. Dalva M. B. (2010). Trans-synaptic EphB2-ephrin-B3 interaction regulates excitatory synapse density by inhibition of postsynaptic MAPK signaling. *Proc. Natl. Acad. Sci. U.S.A.* 107 8830–8835. 10.1073/pnas.0910644107 20410461 PMC2889310

[B86] Miller A. J. Page R. A. Bernal X. E. (2018). Exploratory behavior of a native anuran species with high invasive potential. *Anim. Cogn.* 21 55–65. 10.1007/s10071-017-1138-y 29030724

[B87] Minatohara K. Akiyoshi M. Okuno H. (2016). Role of immediate-early genes in synaptic plasticity and neuronal ensembles underlying the memory trace. *Front. Mol. Neurosci.* 8:78. 10.3389/fnmol.2015.00078 26778955 PMC4700275

[B88] Mitchell M. D. McCormick M. I. (2013). Ontogenetic differences in chemical alarm cue production determine antipredator responses and learned predator recognition. *Behav. Ecol. Sociobiol.* 67 1123–1129. 10.1007/s00265-013-1537-2

[B89] Mokin M. Keifer J. (2005). Expression of the immediate-early gene–encoded protein Egr-1 (zif268) during in vitro classical conditioning. *Learn. Mem.* 12 144–149. 10.1101/lm.87305 15805312 PMC1074332

[B90] Mukherjee A. Williams D. W. (2017). More alive than dead: non-apoptotic roles for caspases in neuronal development, plasticity and disease. *Cell Death Differ.* 24 1411–1421. 10.1038/cdd.2017.64 28644437 PMC5520460

[B91] Nestler E. J. (2015). ΔFosB: a transcriptional regulator of stress and antidepressant responses. *Eur. J. Pharmacol.* 753 66–72. 10.1016/j.ejphar.2014.10.034 25446562 PMC4380559

[B92] Neufeld G. Cohen T. Shraga N. Lange T. Kessler O. Herzog Y. (2002). The neuropilins multifunctional semaphorin and VEGF receptors that modulate axon guidance and angiogenesis. *Trends Cardiovasc. Med.* 12 13–19. 10.1016/S1050-1738(01)00140-2 11796239

[B93] Nolt M. J. Lin Y. Hruska M. Murphy J. Sheffler-Colins S. I. Kayser M. S. (2011). EphB controls NMDA receptor function and synaptic targeting in a subunit-specific manner. *J. Neurosci.* 31 5353–5364. 10.1523/JNEUROSCI.0282-11.2011 21471370 PMC3147026

[B94] Okazaki N. Yan J. Yuasa S. Ueno T. Kominami E. Masuho Y. (2000). Interaction of the Unc-51-like kinase and microtubule-associated protein light chain 3 related proteins in the brain: possible role of vesicular transport in axonal elongation. *Mol. Brain Res.* 85 1–12. 10.1016/S0169-328X(00)00218-7 11146101

[B95] Pagni M. Ioannidis V. Cerutti L. Zahn-Zabal M. Jongeneel C. V. Hau J. (2007). MyHits: improvements to an interactive resource for analyzing protein sequences. *Nucleic Acids Res.* 35 W433–W437. 10.1093/nar/gkm352 17545200 PMC1933190

[B96] Patzke C. Acuna C. Giam L. R. Wernig M. Südhof T. C. (2016). Conditional deletion of *L1CAM* in human neurons impairs both axonal and dendritic arborization and action potential generation. *J. Exp. Med.* 213 499–515. 10.1084/jem.20150951 27001749 PMC4821644

[B97] Perrotti L. I. Hadeishi Y. Ulery P. G. Barrot M. Monteggia L. Duman R. S. (2004). Induction of FosB in reward-related brain structures after chronic stress. *J. Neurosci.* 24 10594–10602. 10.1523/JNEUROSCI.2542-04.2004 15564575 PMC6730117

[B98] Perry C. J. Barron A. B. Cheng K. (2013). Invertebrate learning and cognition: relating phenomena to neural substrate: invertebrate learning and cognition. *WIREs Cogn. Sci.* 4 561–582. 10.1002/wcs.1248 26304245

[B99] Pinaud R. (2004). Experience-dependent immediate early gene expression in the adult central nervous system: evidence from enriched-environment studies. *Int. J. Neurosci.* 114 321–333. 10.1080/00207450490264142 14754658

[B100] Pinaud R. Fortes A. F. Lovell P. Mello C. V. (2006). Calbindin-positive neurons reveal a sexual dimorphism within the songbird analogue of the mammalian auditory cortex. *J. Neurobiol.* 66 182–195. 10.1002/neu.20211 16288476

[B101] Plath N. Ohana O. Dammermann B. Errington M. L. Schmitz D. Gross C. (2006). Arc/Arg3.1 is essential for the consolidation of synaptic plasticity and memories. *Neuron* 52 437–444. 10.1016/j.neuron.2006.08.024 17088210

[B102] Poliak S. Gollan L. Martinez R. Custer A. Einheber S. Salzer J. L. (1999). Caspr2, a new member of the neurexin superfamily, is localized at the juxtaparanodes of myelinated axons and associates with K+ channels. *Neuron* 24 1037–1047. 10.1016/S0896-6273(00)81049-1 10624965

[B103] Ramsay Z. J. Ikura J. Laberge F. (2013). Modification of a prey catching response and the development of behavioral persistence in the fire-bellied toad (*Bombina orientalis*). *J. Comp. Psychol.* 127 399–411. 10.1037/a0032059 23668694

[B104] Rao J. N. Madasu Y. Dominguez R. (2014). Mechanism of actin filament pointed-end caing by tropomodulin. *Science* 345 463–467. 10.1126/science.1256159 25061212 PMC4367809

[B105] Riccomagno M. M. Kolodkin A. L. (2015). Sculpting neural circuits by axon and dendrite pruning. *Annu. Rev. Cell Dev. Biol.* 31 779–805. 10.1146/annurev-cellbio-100913-013038 26436703 PMC4668927

[B106] Roberts A. Pachter L. (2013). Streaming fragment assignment for real-time analysis of sequencing experiments. *Nat. Methods* 10 71–73. 10.1038/nmeth.2251 23160280 PMC3880119

[B107] Roof D. J. Hayes A. Adamian M. Chishti A. H. Li T. (1997). Molecular characterization of abLIM, a novel actin-binding and double zinc finger protein. *J. Cell Biol.* 138 575–588. 10.1083/jcb.138.3.575 9245787 PMC2141644

[B108] Roth W. Kermer P. Krajewska M. Welsh K. Davis S. Krajewski S. (2003). Bifunctional apoptosis inhibitor (BAR) protects neurons from diverse cell death pathways. *Cell Death Differ.* 10 1178–1187. 10.1038/sj.cdd.4401287 14502241

[B109] Rudy J. W. (2014). *The Neurobiology of Learning and Memory*, 2nd Edn Sunderland, MA: Sinauer Associates, Inc.

[B110] Rudy J. W. (2015a). Actin dynamics and the evolution of the memory trace. *Brain Res.* 1621 17–28. 10.1016/j.brainres.2014.12.007 25498985

[B111] Rudy J. W. (2015b). Variation in the persistence of memory: an interplay between actin dynamics and AMPA receptors. *Brain Res.* 1621 29–37. 10.1016/j.brainres.2014.12.009 25511990

[B112] Schaefer N. Rotermund C. Blumrich E.-M. Lourenco M. V. Joshi P. Hegemann R. U. (2017). The malleable brain: plasticity of neural circuits and behavior - a review from students to students. *J. Neurochem.* 142 790–811. 10.1111/jnc.14107 28632905

[B113] Schaffer A. E. Breuss M. W. Caglayan A. O. Al-Sanaa N. Al-Abdulwahed H. Y. Kaymakçalan H. (2018). Biallelic loss of human CTNNA2, encoding αN-catenin, leads to ARP2/3 complex overactivity and disordered cortical neuronal migration. *Nat. Genet.* 50 1093–1101. 10.1038/s41588-018-0166-0 30013181 PMC6072555

[B114] Schmajuk N. A. Segura E. T. Reboreda J. C. (1980). Aetitive conditioning and discriminatory learning in toads. *Behav. Neural Biol.* 28 392–397. 10.1016/s0163-1047(80)91698-2 6773517

[B115] Schuldiner O. Yaron A. (2015). Mechanisms of developmental neurite pruning. *Cell. Mol. Life Sci.* 72 101–119. 10.1007/s00018-014-1729-6 25213356 PMC5086088

[B116] Session A. M. Uno Y. Kwon T. Chapman J. A. Toyoda A. Takahashi S. (2016). Genome evolution in the allotetraploid frog *Xenopus laevis*. *Nature* 538 336–343. 10.1038/nature19840 27762356 PMC5313049

[B117] Shomrat T. Turchetti-Maia A. L. Stern-Mentch N. Basil J. A. Hochner B. (2015). The vertical lobe of cephalopods: an attractive brain structure for understanding the evolution of advanced learning and memory systems. *J. Comp. Physiol. A* 201 947–956. 10.1007/s00359-015-1023-6 26113381

[B118] Sigrist C. J. A. (2002). PROSITE: a documented database using patterns and profiles as motif descriptors. *Brief. Bioinform.* 3 265–274. 10.1093/bib/3.3.265 12230035

[B119] Simon D. J. Weimer R. M. McLaughlin T. Kallop D. Stanger K. Yang J. (2012). A caspase cascade regulating developmental axon degeneration. *J. Neurosci.* 32 17540–17553. 10.1523/JNEUROSCI.3012-12.2012 23223278 PMC3532512

[B120] Sokolowski M. B. (2001). Drosophila: genetics meets behaviour. *Nat. Rev. Genet.* 2 879–890. 10.1038/35098592 11715043

[B121] Squire L. Berg D. Bloom F. E. Du Lac S. Ghosh A. Spitzer N. C. (eds) (2012). *Fundamental Neuroscience.* Cambridge, MA: Academic Press.

[B122] Stewart M. G. Harrison E. Rusakov D. A. Richter-Levin G. Maroun M. (2000). Re-structuring of synapses 24 hours after induction of long-term potentiation in the dentate gyrus of the rat hiocampus in vivo. *Neuroscience* 100 221–227. 10.1016/S0306-4522(00)00295-5 11008162

[B123] Takei K. Oka Y. Satou M. Ueda K. (1987). Distribution of motoneurons involved in the prey-catching behavior in the Japanese toad, Bufo japonicus. *Brain Res.* 410 395–400. 10.1016/0006-8993(87)90346-5 3594249

[B124] Takimoto M. Hamada T. (2014). Acute exercise increases brain region-specific expression of MCT1, MCT2, MCT4, GLUT1, and COX IV proteins. *J. Alied Physiol.* 116 1238–1250. 10.1152/jalphysiol.01288.2013 24610532

[B125] Thomas P. D. (2003). PANTHER: a browsable database of gene products organized by biological function, using curated protein family and subfamily classification. *Nucleic Acids Res.* 31 334–341. 10.1093/nar/gkg115 12520017 PMC165562

[B126] Um J. W. Choii G. Park D. Kim D. Jeon S. Kang H. (2016). IQ motif and SEC7 domain-containing protein 3 (IQSEC3) interacts with gephyrin to promote inhibitory synapse formation. *J. Biol. Chem.* 291 10119–10130. 10.1074/jbc.M115.712893 27002143 PMC4858964

[B127] Um J. W. Pramanik G. Ko J. S. Song M.-Y. Lee D. Kim H. (2014). Calsyntenins function as synaptogenic adhesion molecules in concert with neurexins. *Cell Rep.* 6 1096–1109. 10.1016/j.celrep.2014.02.010 24613359 PMC4101519

[B128] Valles A. Boender A. J. Gijsbers S. Haast R. A. M. Martens G. J. M. de Weerd P. (2011). Genomewide analysis of rat barrel cortex reveals time- and layer-specific mRNA expression changes related to experience-dependent plasticity. *J. Neurosci.* 31 6140–6158. 10.1523/JNEUROSCI.6514-10.2011 21508239 PMC6632955

[B129] Velho T. A. F. Mello C. V. (2008). Synapsins are late activity-induced genes regulated by birdsong. *J. Neurosci.* 28 11871–11882. 10.1523/JNEUROSCI.2307-08.2008 19005052 PMC2610538

[B130] Wada K. Howard J. T. McConnell P. Whitney O. Lints T. Rivas M. V. (2006). A molecular neuroethological aroach for identifying and characterizing a cascade of behaviorally regulated genes. *Proc. Natl. Acad. Sci. U.S.A.* 103 15212–15217. 10.1073/pnas.0607098103 17018643 PMC1622802

[B131] Wang H. Sawai A. Toji N. Sugioka R. Shibata Y. Suzuki Y. (2019). Transcriptional regulatory divergence underpinning species-specific learned vocalization in songbirds. *PLoS Biol.* 17:e3000476. 10.1371/journal.pbio.3000476 31721761 PMC6853299

[B132] Weiss J. L. Hui H. Burgoyne R. D. (2010). Neuronal calcium sensor-1 regulation of calcium channels, secretion, and neuronal outgrowth. *Cell. Mol. Neurobiol.* 30 1283–1292. 10.1007/s10571-010-9588-7 21104311 PMC11498851

[B133] Wolman M. A. Liu Y. Tawarayama H. Shoji W. Halloran M. C. (2004). Repulsion and attraction of axons by Semaphorin3D are mediated by different neuropilins in vivo. *J. Neurosci.* 24 8428–8435. 10.1523/JNEUROSCI.2349-04.2004 15456815 PMC6729895

[B134] Xu N. J. Henkemeyer M. (2009). Ephrin-B3 reverse signaling through Grb4 and cytoskeletal regulators mediates axon pruning. *Nat. Neurosci.* 12 268–276. 10.1038/nn.2254 19182796 PMC2661084

[B135] Yu G. Yang J. Zhang M. Rao D. (2007). Phylogenetic and systematic study of the genus *Bombina* (Amphibia: Anura: Bombinatoridae): new insights from molecular data. *J. Herpetol.* 41 365–377. 10.1670/0022-1511(2007)41

[B136] Zhang H. Xu Q. Krajewski S. Krajewska M. Xie Z. Fuess S. (2000). BAR: an apoptosis regulator at the intersection of caspases and Bcl-2 family proteins. *Proc. Natl. Acad. Sci. U.S.A.* 97 2597–2602. 10.1073/pnas.97.6.2597 10716992 PMC15974

[B137] Zhang J. Gao J.-X. Salojin K. Shao Q. Grattan M. Meagher C. (2000). Regulation of FAS ligand expression during activation-induced cell death in T Cells by p38 mitogen-activated protein kinase and C-Jun Nh 2 -Terminal Kinase. *J. Exp. Med.* 191 1017–1030. 10.1084/jem.191.6.1017 10727463 PMC2193110

[B138] Zhao Y. Fung C. Shin D. Shin B.-C. Thamotharan S. Sankar R. (2010). Neuronal glucose transporter isoform 3 deficient mice demonstrate features of autism spectrum disorders. *Mol. Psychiatry* 15 286–299. 10.1038/mp.2009.51 19506559 PMC4208914

