# Supplementary material for: Temporal Profile of Brain Gene Expression After Prey Catching Conditioning in an Anuran Amphibian
Source: Front Neurosci. 2020 Jan 14;13:1407. doi: 10.3389/fnins.2019.01407 (PMC6971186; doi:10.3389/fnins.2019.01407)
Supplement: Supplementary file 1 [file Table_1.docx]

**Supplementary material**

*Section 1: Figures & Tables*

**Table S1:** *De novo* transcriptome alignment quality statistics. Raw RNA-seq reads were cleaned using the Trimmomatic software suite. Raw reads were aligned back against the transcriptome using the Bowtie2 software suite. Alignment statistics and overall alignment rates are presented for each toad.

|  | **Raw reads** | **Clean reads** | **%Unaligned** | **%Aligned concordantly once** | **%Aligned concordantly**  **> 1** | **Overall alignment rate%** |
| --- | --- | --- | --- | --- | --- | --- |
| **Control 1** | 22312799 | 21357615 | 18.79 | 23.06 | 58.15 | 93.26 |
| **Control 2** | 22185273 | 21428734 | 19.20 | 23.16 | 57.64 | 93.29 |
| **Control 3** | 20177889 | 19447444 | 18.65 | 23.16 | 58.19 | 93.27 |
| **2-Hour 1** | 23226803 | 22400117 | 18.48 | 22.96 | 58.56 | 93.47 |
| **2-Hour 2** | 21404318 | 20660556 | 18.70 | 22.84 | 58.45 | 93.35 |
| **2-Hour 3** | 23395990 | 22673001 | 18.97 | 23.14 | 57.89 | 93.28 |
| **4-Hour 1** | 22599349 | 21847763 | 18.77 | 23.20 | 58.03 | 93.27 |
| **4-Hour 2** | 22394847 | 21684270 | 19.34 | 23.03 | 57.62 | 93.24 |
| **4-Hour 3** | 21985162 | 21376973 | 19.59 | 23.29 | 57.12 | 93.33 |
| **24-Hour 1** | 22079482 | 21302679 | 18.48 | 23.23 | 58.29 | 93.43 |
| **24-Hour 2** | 23178754 | 22332319 | 18.30 | 23.37 | 58.34 | 93.49 |
| **24-Hour 3** | 23984646 | 23018894 | 18.39 | 23.31 | 58.30 | 93.25 |

**Table S2**: *De novo* transcriptome assembly statistics. The Trinity software package was used to assemble a transcriptome based on ~250 million paired end reads utilizing De Bruijn graph-based transcript assembly. Trinity assembles multiple isoforms for each assembled ‘gene’, row 1 represents the statistics for all transcripts assembled, while row 2 represents the statistics for only the longest assembled isoform. N50 represents the minimum length of at least 50% of assembled transcripts.

|  | **Total Base Pairs** | **Total transcripts** | **N50** | **Average length** | **Median length** | **GC%** | **Total genes** |
| --- | --- | --- | --- | --- | --- | --- | --- |
| **All transcripts** | 656704220 | 1236332 | 668 | 531.17 | 311 | 39.59 | 714091 |
| **Longest isoform** | 339551472 | - | 552 | 475.5 | 312 | - | - |

**Table S3a**: Gene ontology functional group enrichment of differentially expressed genes in the *Early* co-expression profile (2 h post-training) in comparison to controls.

.

| **GO category** | **Functional group** | **Regulation** | **Gene count** |
| --- | --- | --- | --- |
| Biological process | GO:0006355~regulation of transcription, DNA-templated | UP | 14 |
| Biological process | GO:0007015~actin filament organization | UP | 3 |
| Biological process | GO:0048167~regulation of synaptic plasticity | UP | 3 |
| Biological process | GO:0030036~actin cytoskeleton organization | UP | 3 |
| Biological process | GO:0007411~axon guidance | UP | 3 |
| Biological process | GO:0006810~transport | UP | 5 |
| Biological process | GO:0015031~protein transport | UP | 4 |
| Biological process | GO:0031175~neuron projection development | Down | 4 |
| Biological process | GO:0043410~positive regulation of MAPK cascade | Down | 3 |
| Biological process | GO:0000226~microtubule cytoskeleton organization | Down | 3 |
| Cell component | GO:0005622~intracellular | UP | 12 |
| Cell component | GO:0045254~pyruvate dehydrogenase complex | UP | 2 |
| Cell component | GO:0043025~neuronal cell body | UP | 6 |
| Cell component | GO:0005913~cell-cell adherens junction | UP | 4 |
| Cell component | GO:0030426~growth cone | UP | 3 |
| Cell component | GO:0005925~focal adhesion | UP | 5 |
| Cell component | GO:0030424~axon | UP | 4 |
| Cell component | GO:0005634~nucleus | UP | 31 |
| Cell component | GO:0005886~plasma membrane | Down | 30 |
| Cell component | GO:0030027~lamellipodium | Down | 5 |
| Cell component | GO:0005765~lysosomal membrane | Down | 5 |
| Cell component | GO:0005913~cell-cell adherens junction | Down | 5 |
| Cell component | GO:0071556~integral component of lumenal side of endoplasmic reticulum membrane | Down | 3 |
| Cell component | GO:0005796~Golgi lumen | Down | 3 |
| Cell component | GO:0005776~autophagosome | Down | 3 |
| Cell component | GO:0031902~late endosome membrane | Down | 3 |
| Molecular function | GO:0046983~protein dimerization activity | UP | 4 |
| Molecular function | GO:0003676~nucleic acid binding | UP | 7 |
| Molecular function | GO:0003677~DNA binding | UP | 11 |
| Molecular function | GO:0003700~transcription factor activity, sequence-specific DNA binding | UP | 6 |
| Molecular function | GO:0005515~protein binding | Down | 34 |
| Molecular function | GO:0008022~protein C-terminus binding | Down | 4 |

**Table S3b**: Gene ontology functional group enrichment of differentially expressed genes in the *Intermediate* co-expression profile (4 h post-training) in comparison to controls.

| **GO category** | **Functional group** | **Regulation** | **Gene count** |
| --- | --- | --- | --- |
| Biological process | GO:0007411~axon guidance | UP | 5 |
| Biological process | GO:0016337~single organismal cell-cell adhesion | UP | 4 |
| Biological process | GO:0007157~cell-cell adhesion via plasma membrane adhesion molecules | UP | 3 |
| Biological process | GO:0007160~cell-matrix adhesion | UP | 3 |
| Biological process | GO:0007156~homophilic cell adhesion via plasma membrane adhesion molecules | UP | 3 |
| Biological process | GO:0006897~endocytosis | UP | 4 |
| Biological process | GO:0006915~apoptotic process | UP | 4 |
| Biological process | GO:0008152~metabolic process | UP | 3 |
| Biological process | GO:0006355~regulation of transcription, DNA-templated | Down | 15 |
| Biological process | GO:0006351~transcription, DNA-templated | Down | 15 |
| Biological process | GO:0007409~axonogenesis | Down | 4 |
| Biological process | GO:0010976~positive regulation of neuron projection development | Down | 3 |
| Biological process | GO:0046777~protein autophosphorylation | Down | 5 |
| Biological process | GO:0006461~protein complex assembly | Down | 3 |
| Biological process | GO:0016311~dephosphorylation | Down | 3 |
| Biological process | GO:0006541~glutamine metabolic process | Down | 3 |
| Biological process | GO:0006006~glucose metabolic process | Down | 3 |
| Biological process | GO:0008152~metabolic process | Down | 3 |
| Cell component | GO:0005739~mitochondrion | UP | 11 |
| Cell component | GO:0005667~transcription factor complex | UP | 4 |
| Cell component | GO:0030175~filopodium | UP | 3 |
| Cell component | GO:0005813~centrosome | Down | 7 |
| Cell component | GO:0005783~endoplasmic reticulum | Down | 11 |
| Cell component | GO:0005789~endoplasmic reticulum membrane | Down | 10 |
| Cell component | GO:0014069~postsynaptic density | Down | 5 |
| Cell component | GO:0098793~presynapse | Down | 3 |
| Molecular function | GO:0003676~nucleic acid binding | UP | 8 |
| Molecular function | GO:0051287~NAD binding | Down | 3 |
| Molecular function | GO:0000287~magnesium ion binding | Down | 5 |

**Table S3c**: Gene ontology functional group enrichment of differentially expressed genes in the *Late* co-expression profile (24 h post-training) in comparison to controls.

| **GO category** | **Functional group** | **Regulation** | **Gene count** |
| --- | --- | --- | --- |
| Biological process | GO:0006355~regulation of transcription, DNA-templated | UP | 19 |
| Biological process | GO:0006351~transcription, DNA-templated | UP | 23 |
| Biological process | GO:0043065~positive regulation of apoptotic process | UP | 9 |
| Biological process | GO:0031175~neuron projection development | UP | 6 |
| Biological process | GO:0046326~positive regulation of glucose import | UP | 3 |
| Biological process | GO:0048041~focal adhesion assembly | UP | 3 |
| Biological process | GO:0007015~actin filament organization | UP | 4 |
| Biological process | GO:0007010~cytoskeleton organization | UP | 3 |
| Biological process | GO:0007049~cell cycle | Down | 8 |
| Biological process | GO:0098609~cell-cell adhesion | Down | 6 |
| Biological process | GO:0051017~actin filament bundle assembly | Down | 3 |
| Cell component | GO:0005819~spindle | UP | 5 |
| Cell component | GO:0016363~nuclear matrix | UP | 4 |
| Cell component | GO:0030496~midbody | Down | 6 |
| Cell component | GO:0005913~cell-cell adherens junction | Down | 8 |
| Cell component | GO:0009897~external side of plasma membrane | Down | 6 |
| Cell component | GO:0030133~transport vesicle | Down | 4 |
| Cell component | GO:0031902~late endosome membrane | Down | 4 |
| Molecular function | GO:0003723~RNA binding | UP | 12 |
| Molecular function | GO:0003677~DNA binding | UP | 23 |
| Molecular function | GO:0003779~actin binding | UP | 8 |
| Molecular function | GO:0046872~metal ion binding | UP | 33 |
| Molecular function | GO:0004519~endonuclease activity | UP | 5 |
| Molecular function | GO:0003964~RNA-directed DNA polymerase activity | UP | 4 |
| Molecular function | GO:0016791~phosphatase activity | UP | 4 |
| Molecular function | GO:0005515~protein binding | Down | 56 |
| Molecular function | GO:0042605~peptide antigen binding | Down | 4 |
| Molecular function | GO:0036459~thiol-dependent ubiquitinyl hydrolase activity | Down | 4 |
| Molecular function | GO:0098641~cadherin binding involved in cell-cell adhesion | Down | 7 |
| Molecular function | GO:0004843~thiol-dependent ubiquitin-specific protease activity | Down | 4 |

**Table S4a**: Details of gene ontology enrichment analysis in *Early* co-expression profile. Enriched GO groups have been grouped by similar function into manually selected non-GO functional categories (*Functional category).* The *Gene ontology functional group* column represents all enriched GO groups included in the associated *functional category.* The *Included in GO analysis column* represents all genes included in the enriched GO groups, separated by up and down regulation. The *Similar function* column represents all genes which were part of non-enriched GO functional groups that fall under the relevant *Functional category*.

| **Functional Category** | **Direction of regulation** | | **Gene ontology functional group** | **Included in GO analysis** | **Similar function** |
| --- | --- | --- | --- | --- | --- |
| Transcriptional regulation | Up | GO:0006355~regulation of transcription, DNA-templated | | Zinc finger protein 614  Krueppel-related zinc finger protein 1  Zinc finger protein 347  Thioredoxin-interacting protein  Oocyte zinc finger protein XlCOF8.4  Zinc finger protein 577  Amyloid-beta A4 precursor protein- binding family B member 3  Neuronal PAS domain-containing protein 4B  Protein furry homolog-like  Zinc finger protein 585B  Early growth response protein 1-B  E3 ubiquitin-protein ligase MYCBP2  Lamina-associated polypeptide 2  Histone-lysine N-methyltransferase PRDM9 | Proto-oncogene c-Fos  Immediate early response gene 2 protein  Transcription factor jun-D  DNA-binding protein inhibitor ID-4  Zinc finger MYM-type protein 1  Zinc finger BED domain-containing protein 4  UAP56-interacting factor  Zinc finger protein 706  Gastrula zinc finger protein XlCGF26.1  Genome polyprotein  LIM domain only protein 7  NHP2-like protein 1  Nuclear factor interleukin-3-regulated protein |
|  | Down | - | | - | Host cell factor 1  Transcription factor 25  Transcription factor Sp2  Zinc finger and BTB domain-containing protein 25  Zinc finger protein GLI4  Zinc finger protein 300 |
| Structural plasticity | Up | GO:0007015~actin filament organization  GO:0030036~actin cytoskeleton organization  GO:0007411~axon guidance  GO:0051965~positive regulation of synapse assembly | | Tropomodulin-3  Tropomodulin-2  Sorbin and SH3 domain-containing protein 2  IQ motif and SEC7 domain-containing protein 3  Protein kinase C and casein kinase substrate in neurons protein 2  ETS translocation variant 1  Neural cell adhésion molecule L1  Ephrin type-B receptor 2  Leucine-rich repeat-containing protein 24 | NCK-interacting protein with SH3 domain  Switch-associated protein 70  Tubulin beta-4B chain  E3 ubiquitin-protein ligase MYCBP2  Protein furry homolog-like  Rho-related GTP-binding protein RhoQ  Stromal cell-derived factor 1  Semaphorin-7A |
|  | Down | - | | - | Ankyrin repeat and SAM domain-containing protein 1A  Annexin A6  Band 4.1-like protein 1  CDC42 small effector protein 2-C  Engulfment and cell motility protein 1  Fasciculation and elongation protein zeta-1  Protein RUFY3  Roundabout homolog 2  Unconventional myosin-X |
| Transport | Up | GO:0006810~transport  GO:0015031~protein transport | | Long-chain fatty acid transport protein 4  Glutamate receptor 1  Ras-related protein Rab-15  Neutral amino acid transporter B(0)  Tropomodulin-3  Cysteine protease ATG4B  Formin-2  Mitochondrial import inner membrane translocase subunit TIM14 | Kinesin-like protein KIF1A  Transmembrane emp24 domain-containing protein 2  Dynactin subunit 6  Equilibrative nucleoside transporter 1  Multivesicular body subunit 12A |
|  | Down | - | | - | - |
| Apoptosis | Up | - | | - | BTB/POZ domain-containing protein 10  Dedicator of cytokinesis protein 1  E3 ubiquitin-protein ligase RNF130  Palmitoyl-protein thioesterase 1 |
|  | Down | - | | - | Protein AF1q  Protein FAN  Annexin A6  Engulfment and cell motility protein 1 |
| Process genesis | Up | - | | - | - |
|  | Down | GO:0031175~neuron projection development | | Serine/threonine-protein kinase ULK1  Ephrin type-A receptor 8  Dedicator of cytokinesis protein 7  F-actin-capping protein subunit beta  Pericentrin  Gamma-aminobutyric acid receptor-associated protein | Reticulon-4  Neuronal tyrosine-phosphorylated phosphoinositide-3-kinase adapter 1  Pre-B-cell leukemia transcription factor 3  Protein phosphatase Slingshot homolog 2  Tripartite motif-containing protein 3  Ubiquitin conjugation factor E4 B  Cadherin-2  Catenin beta-1  Glypican-4 |

**Table S4b**: Details of gene ontology enrichment analysis in *Intermediate* co-expression profile. Enriched GO groups have been grouped by similar function into manually selected non-GO functional categories (*Functional category).* The *Gene ontology functional group* column represents all enriched GO groups included in the associated *functional category.* The *Included in GO analysis column* represents all genes included in the enriched GO groups, separated by up and down regulation. The *Similar function* column represents all genes which were part of non-enriched GO functional groups that fall under the relevant *Functional category*.

| **Functional Category** | **Direction of regulation** | **Gene ontology functional group** | **Included in GO analysis** | **Similar function** |
| --- | --- | --- | --- | --- |
| Transcriptional regulation | Up | - | - | Histone deacetylase 9  Oocyte zinc finger protein XlCOF6.1  PIH1 domain-containing protein 1  Protein max  RNA-binding protein 14  SAP domain-containing ribonucleoprotein  Signal transducer and activator of transcription 1-alpha/beta  Transcriptional regulator ATRX  Transcriptional-regulating factor 1  X-box-binding protein 1 |
|  | Down | GO:0006355~regulation of transcription, DNA-templated  GO:0006351~transcription, DNA-templated | Krueppel-related zinc finger protein 1  Zinc finger protein 341  Serine/threonine-protein kinase NLK  Period circadian protein homolog 2  Zinc finger and SCAN domain-containing protein 20  Serine/threonine-protein kinase/endoribonuclease IRE1  Histone-lysine N-methyltransferase 2D  Protein furry homolog-like  Circadian-associated transcriptional repressor  KRAB domain-containing protein 4  Lamina-associated polypeptide 2, isoform alpha  Hepatoma-derived growth factor  Zinc finger protein 160  KH domain-containing, RNA-binding, signal transduction-associated protein 3  Histone deacetylase 5  Ataxin-7-like protein 3  Zinc finger and BTB domain-containing protein 18 | Vacuolar protein sorting-associated protein 72 homolog  Chorion transcription factor Cf2  SAP_PIG Saposin-B-Val |
| Structural plasticity | Up | GO:0007411~axon guidance  GO:0016337~single organismal cell-cell adhesion  GO:0007157~heterophilic cell-cell adhesion via plasma membrane cell adhesion molecules  GO:0007160~cell-matrix adhesion  GO:0007156~homophilic cell adhesion via plasma membrane adhesion molecules | Dihydropyrimidinase-related protein 2  Neuropilin-2  Nuclear receptor subfamily 4 group A member 3  Protein enabled homolog  Ephrin-A5  Polycystin-1  Disks large homolog 5  Vascular cell adhesion protein 1  Galectin-9  Protocadherin gamma-B5  Protocadherin gamma-C5 | Rho-related GTP-binding protein Rho6  Adenylyl cyclase-associated protein 2  Calpain-1 catalytic subunit  Mitogen-activated protein kinase 9  Semaphorin-3D  Protein Tob1  Coiled-coil domain-containing protein 134  Intraflagellar transport protein 27 homolog  Probable 28S rRNA (cytosine-C(5))-methyltransferase |
|  | Down | - | - | Adhesion G protein-coupled receptor L3  Band 4.1-like protein 1  Guanine nucleotide exchange C9orf72  Kelch-like protein 17  Neurexin-2  Semaphorin-7A |
| Transport | Up | GO:0006897~endocytosis | Extended synaptotagmin-2-A  Dihydropyrimidinase-related protein 2  Low-density lipoprotein receptor-related protein 5  SH3-containing GRB2-like protein 3-interacting protein 1 | Synaptotagmin-C  Prenylated Rab acceptor protein 1  Dynactin subunit 6  Serine/threonine-protein kinase 11-interacting protein  TOM1-like protein 2 |
|  | Down | - | - | Adhesion G protein-coupled receptor L2  ADP-ribosylation factor 1  Calcium-dependent secretion activator 2  Conserved oligomeric Golgi complex subunit 1  Importin subunit alpha-3  Kinesin light chain 1  Kinesin-associated protein 3  Syntabulin  Syntaxin-6  Transmembrane emp24 domain-containing protein 4 |
| Apoptosis | Up | GO:0006915~apoptotic process | Fibroblast growth factor receptor 2  FAS-associated factor 1  Death domain-associated protein 6  Ephrin-A5 | Atrophin-1  E3 ubiquitin-protein ligase RNF152  SID1 transmembrane family member 2 |
|  | Down | - | - | RAC-alpha serine/threonine-protein kinase  Cleft lip and palate transmembrane protein 1-like protein |
| Process genesis | Up | - | - | BICD family-like cargo adapter 1  Disks large homolog 5  Fibroblast growth factor receptor 2  MICAL-like protein 2  Mitogen-activated protein kinase 9  Zinc finger and BTB domain-containing protein 16-A |
|  | Down | GO:0007409~axonogenesis  GO:0010976~positive regulation of neuron projection development | Catenin alpha-2  Serine/threonine-protein kinase BRSK2  Clathrin coat assembly protein AP180  Protein-tyrosine kinase 2-beta  Membrane-associated guanylate kinase, WW and PDZ domain-containing protein 2  Glia-derived nexin  Protein furry homolog-like  BICD family-like cargo adapter 1 | SUN domain-containing protein 1  Ankyrin repeat and SAM domain-containing protein 1A  Neuronal PAS domain-containing protein 4  V-type proton ATPase catalytic subunit A |

**Table S4c**: Details of gene ontology enrichment analysis in *Late* co-expression profile. Enriched GO groups have been grouped by similar function into manually selected non-GO functional categories (*Functional category).* The *Gene ontology functional group* column represents all enriched GO groups included in the associated *functional category.* The *Included in GO analysis column* represents all genes included in the enriched GO groups, separated by up and down regulation. The *Similar function* column represents all genes which were part of non-enriched GO functional groups that fall under the relevant *Functional category*.

| **Functional Category** | **Direction of regulation** | **Gene ontology functional group** | **Included in GO analysis** | **Similar function** |
| --- | --- | --- | --- | --- |
| Transcriptional regulation | Up | GO:0006355~regulation of transcription, DNA-templated  GO:0006351~transcription, DNA-templated | Transcriptional-regulating factor 1  Neuronal PAS domain-containing protein 3  Krueppel-related zinc finger protein 1  PHD finger protein 20  Zinc finger protein GLI4  YEATS domain-containing protein 4  Zinc finger protein 84  Histone-lysine N-methyltransferase 2D  Zinc finger protein 22  Cyclin-H  Zinc finger protein 300  Cyclin-L2  Putative zinc finger protein 826  Zinc finger protein neuro-d4  PR domain zinc finger protein 15  Metabotropic glutamate receptor 5  Lamina-associated polypeptide 2, isoform alpha  Chromodomain-helicase-DNA-binding protein 9  Leucine-rich PPR motif-containing protein, mitochondrial  General transcription factor II-I  PAX3- and PAX7-binding protein 1  Myelin transcription factor 1  Actin-binding LIM protein 2  SWI/SNF-related matrix-associated actin-dependent regulator of – chromatin subfamily D member 2  Chromobox protein homolog 3  Death-inducer obliterator 1 | DNA-directed RNA polymerase II subunit RPB7  Eukaryotic translation initiation factor 4 gamma 2  Gastrula zinc finger protein XlCGF49.1  Mediator of RNA polymerase II transcription subunit 16  Protein FAM50A  Transcription elongation regulator 1 |
|  | Down | -- | - | CCAAT/enhancer-binding protein gamma  Oocyte zinc finger protein XlCOF26  Replication factor C subunit 1  Zinc finger protein 182 |
| Apoptosis | Up | GO:0043065~positive regulation of apoptotic process | General transcription and DNA repair factor IIH helicase subunit XPB  Protein FAN  Transcription factor AP-4  Slit homolog 2 protein  Protein kinase C delta type  Rho guanine nucleotide exchange factor 12  Dual specificity protein phosphatase 6  Programmed cell death protein 4  Death-inducer obliterator 1 | N-alpha-acetyltransferase 35_ NatC auxiliary subunit  Next to BRCA1 gene 1 protein  RELT-like protein 2  TIP41-like protein  Calpain-1 catalytic subunit  GPI transamidase component PIG-T |
|  | Down | - | - | V-set domain-containing T-cell activation inhibitor 1  Ubiquitin-conjugating enzyme E2 Z  RNA-binding protein 5  DnaJ homolog subfamily A member 1  Death domain-associated protein 6  Bifunctional apoptosis regulator  B-cell lymphoma/leukemia 11B |
| Process genesis | Up | GO:0031175~neuron projection development | Probable E3 ubiquitin-protein ligase HERC1  Contactin-associated protein-like 2  Protein furry homolog  Contactin-associated protein 1  Brain-specific angiogenesis inhibitor 1-associated protein 2  AP-2 complex subunit alpha-1 | Mimecan  Failed axon connections homolog  Cytochrome b-c1 complex subunit 8  Adhesion G protein-coupled receptor B1  Actin-related protein 3  Disabled homolog 1  E3 ubiquitin-protein ligase HECW1  Metabotropic glutamate receptor |
|  | Down | -- | - | H-2 class I histocompatibility antigen K-K alpha chain  Microtubule-associated protein 2  Tyrosine-protein kinase Lyn |
| Structural plasticity | Up | GO:0007015~actin filament organization  GO:0048041~focal adhesion assembly  GO:0007010~cytoskeleton organization  hsa04360:Axon guidance (KEGG pathway) | Sorbin and SH3 domain-containing protein 1  WASP homolog-associated protein with actin, membranes and microtubules  Epsin-1  Serine/threonine-protein kinase MRCK alpha  Adenylyl cyclase-associated protein 2  Contactin-associated protein 1  Actin-binding LIM protein 2  Rho guanine nucleotide exchange factor 12 | Semaphorin-6A  Protocadherin gamma-B1  Protocadherin-17  ETS translocation variant 1  Slit homolog 2 protein  Protein enabled homolog  Adenylyl cyclase-associated protein 2  C-C chemokine receptor type 9  Connector enhancer of kinase suppressor of ras 2  Nuclear protein MDM1  Spectrin alpha chain non-erythrocytic 1  Tubulin alpha-1 chain  WASP homolog-associated protein with actin membranes and microtubules |
|  | Down | GO:0098609~cell-cell adhesion  GO:0051017~actin filament bundle assembly | EH domain-containing protein 4  Lamina-associated polypeptide 2, isoforms alpha/zeta  Ubiquitin carboxyl-terminal hydrolase 8  Rho GTPase-activating protein 1  Lamina-associated polypeptide 2, isoform alpha  TBC1 domain family member 10A  Epidermal growth factor receptor kinase substrate 8  Switch-associated protein 70  Beta-adducin | Protocadherin gamma-B1  Aggrecan core protein  Reticulon-4  CaM kinase-like vesicle-associated protein  CKLF-like MARVEL transmembrane domain-containing protein 5  E3 ubiquitin-protein ligase SMURF1  Mammalian ependymin-related protein 1  H-2 class I histocompatibility antigen_ K-K alpha chain  Oncostatin-M-specific receptor subunit beta  Protein NDNF  Tyrosine-protein kinase Lyn |
| Transport | Up | - | - | ADP-ribosylation factor GTPase-activating protein 1  BTB/POZ domain-containing protein 9  Epsin-1  Mitochondrial import inner membrane translocase subunit TIM44  PEX5-related protein  Rab3 GTPase-activating protein non-catalytic subunit  Ras-related protein Rab-36  Synaptic vesicle glycoprotein 2C  Synaptotagmin-C  TBC1 domain family member 17  Transposable element Tcb1 transposase  Tetratricopeptide repeat protein 7B  Transmembrane emp24 domain-containing protein 2  t-SNARE domain-containing protein 1  Tubulin alpha-1 chain  Tumor protein p63-regulated gene 1-like protein  Tyrosine-protein kinase transforming protein erbB  Ubiquilin-1  Ubiquitin carboxyl-terminal hydrolase CYLD  Ubiquitin-like-specific protease ESD4  UPF0472 protein C16orf72 homolog  Uridine-cytidine kinase 2  Usher syndrome type-1G protein homolog  Vacuolar protein sorting-associated protein 8 homolog |
|  | Down | - | - | Biogenesis of lysosome-related organelles complex 1 subunit 5  Charged multivesicular body protein 4b  EH domain-containing protein 4  FK506-binding protein 15  Golgi SNAP receptor complex member 2  Kinesin light chain 4  Nephrocystin-1  Ran-binding protein 3  Zinc finger FYVE domain-containing protein 16 |

**Table S5:** Average transcripts per million (TPM) for all genes in enriched KEGG pathways. Pathway analysis was performed for all differentially expressed transcripts using the Kyoto Encyclopedia of Genes and Genomes (KEGG) database. Only values for transcripts included in significantly enriched pathways (p>=0.05; Fisher’s exact test) are reported. TPM values were obtained from the *eXpress* read alignment tool and were averaged across replicates (+/- StErr).

|  |  |  | Average TPM's (+/- StErr) | | | | | | | |
| --- | --- | --- | --- | --- | --- | --- | --- | --- | --- | --- |
| KEGG pathway | Fisher’s exact | Gene | Control | +/- StErr | 2 Hours | +/- StErr | 4 Hours | +/- StErr | 24 Hours | +/- StErr |
| hsa04360: Axon guidance | P = 0.0006 | Neural cell adhesion molecule L1 | **21.72086** | **0.577645** | **47.80975** | **8.923301** | **28.56571** | **4.435366** | **27.37** | **0.768626** |
|  |  | Netrin-1 | **0.31262** | **0.023406** | **0.02488** | **0.009018** | **0.10334** | **0.067989** | **0.12457** | **0.024612** |
|  |  | Ephrin type-A receptor 8 | **0.28633** | **0.024589** | **0.09678** | **0.001917** | **0.16925** | **0.033527** | **0.20916** | **0.051286** |
|  |  | Slit homolog 2 protein | **0.01212** | **0.008914** | **0.19449** | **0.104754** | **0.33571** | **0.140446** | **0.22953** | **0.028342** |
|  |  | Ephrin type-B receptor 2 | **0.00005** | **4.04E-05** | **0.88853** | **0.200265** | **0.56031** | **0.243982** | **0.60588** | **0.309004** |
|  |  | Rho-related GTP-binding protein Rho6 | **0.07707** | **0.060425** | **3.3686** | **2.306526** | **7.74034** | **3.315619** | **0.15583** | **0.063572** |
|  |  | Actin-binding LIM protein 2 | **0.00678** | **0.005156** | **0.00245** | **0.001501** | **0.65723** | **0.533905** | **1.12901** | **0.451702** |
|  |  | Rho guanine nucleotide exchange factor 12 | **0.55294** | **0.09405** | **0.74728** | **0.233405** | **0.7366** | **0.229872** | **1.53546** | **0.220276** |
| hsa05168: Herpes simplex infection | P = 0.00059 | Tyrosine-protein phosphatase non-receptor type 11 | **0.00739** | **0.000797** | **1.83417** | **0.235172** | **1.0762** | **0.412534** | **0.98797** | **0.400023** |
|  |  | Proto-oncogene c-Fos | **0.06501** | **0.012142** | **1.32774** | **0.746826** | **0.29398** | **0.099408** | **0.14732** | **0.053434** |
|  |  | Class I histocompatibility antigen_ F10 alpha chain | **11.87696** | **1.570289** | **4.34494** | **1.252394** | **8.49862** | **3.448259** | **3.80686** | **0.115395** |
|  |  | Period circadian protein homolog 2 | **1.05342** | **0.412188** | **0.65869** | **0.488398** | **0.06172** | **0.019093** | **0.64332** | **0.515008** |
|  |  | Class II histocompatibility antigen_ B-L beta chain | **17.72676** | **2.697334** | **17.11599** | **2.488512** | **21.82179** | **2.873311** | **4.85164** | **0.69498** |
|  |  | Period circadian protein homolog 3 | **0.43127** | **0.030692** | **0.1921** | **0.082059** | **0.21837** | **0.024676** | **0.12388** | **0.030247** |
|  |  | Eukaryotic translation initiation factor 2-alpha kinase 3 | **0.45793** | **0.163598** | **0.03466** | **0.014936** | **0.00394** | **0.003175** | **0.09251** | **0.041979** |
|  |  | HLA class II histocompatibility antigen DRB1-4 beta chain | **3.03008** | **1.251505** | **0.10034** | **0.081926** | **1.25425** | **0.696452** | **0.10374** | **0.084703** |
|  |  | Probable ATP-dependent RNA helicase DDX58 | **3.11627** | **0.872059** | **0.31629** | **0.064282** | **1.09502** | **0.331185** | **0.75971** | **0.559077** |
|  |  | HLA class II histocompatibility antigen DRB1-9 beta chain | **3.97474** | **1.016881** | **2.78^-69^** | **2.90^-52^** | **1.53434** | **1.252781** | **1.60128** | **0.37847** |
|  |  | Death domain-associated protein 6 | **1.02589** | **0.514829** | **0.55315** | **0.319725** | **0.43115** | **0.118576** | **0.0332** | **0.019526** |
| hsa05169: Epstein-Barr virus infection | P =0.047 | HLA class II histocompatibility antigen DRB1-4 beta chain | **3.03008** | **1.251505** | **0.10034** | **0.081926** | **1.25425** | **0.696452** | **0.10374** | **0.084703** |
|  |  | Probable ATP-dependent RNA helicase DDX58 | **3.11627** | **0.872059** | **0.31629** | **0.064282** | **1.09502** | **0.331185** | **0.75971** | **0.559077** |
|  |  | Ectonucleoside triphosphate diphosphohydrolase 3 | **24.412** | **3.125867** | **12.39731** | **0.625853** | **10.44884** | **4.92963** | **10.13298** | **3.370224** |
|  |  | Tyrosine-protein kinase Lyn | **0.55258** | **0.193562** | **0.12118** | **0.096689** | **0.08206** | **0.033048** | **0.00397** | **0.001709** |
| hsa04141: Protein processing in ER | P =0.041 | ER degradation-enhancing alpha-mannosidase-like protein 1 | **0.00031** | **0.000254** | **0.01619** | **0.008747** | **0.4157** | **0.043879** | **0.38385** | **0.180428** |
|  |  | Eukaryotic translation initiation factor 2-alpha kinase 3 | **0.45793** | **0.163598** | **0.03466** | **0.014936** | **0.00394** | **0.003175** | **0.09251** | **0.041979** |
|  |  | E3 ubiquitin-protein ligase RNF5 | **4.23109** | **1.410057** | **5.54343** | **1.407101** | **12.14245** | **1.409173** | **20.92823** | **1.056655** |
|  |  | Mannosyl-oligosaccharide 1_2-alpha-mannosidase IA | **0.91186** | **0.545284** | **0.19286** | **0.157357** | **0.93372** | **0.414451** | **0.00545** | **0.003233** |
|  |  | Serine/threonine-protein kinase/endoribonuclease IRE1 | **0.67872** | **0.307058** | **0.94665** | **0.528506** | **0.00833** | **0.006801** | **1.09581** | **0.698369** |
|  |  | Ubiquilin-1 | **0.03462** | **0.022696** | **0.01627** | **0.006651** | **0.74348** | **0.318178** | **0.81517** | **0.195491** |
| hsa04514:Cell adhesion molecules (CAMs) | P =0.006 | Neural cell adhesion molecule L1 | **21.72086** | **0.577645** | **47.80975** | **8.923301** | **28.56571** | **4.435366** | **27.37** | **0.768626** |
|  |  | HLA class II histocompatibility antigen DRB1-4 beta chain | **3.03008** | **1.251505** | **0.10034** | **0.081926** | **1.25425** | **0.696452** | **0.10374** | **0.084703** |
|  |  | Cadherin-2 | **1.50865** | **0.191143** | **0.36104** | **0.084761** | **1.52535** | **0.582743** | **1.11722** | **0.564787** |
|  |  | Junctional adhesion molecule C | **0.6827** | **0.153777** | **0.12973** | **0.034756** | **0.24049** | **0.117583** | **0.122** | **0.006951** |
|  |  | HLA class II histocompatibility antigen DRB1-9 beta chain | **3.97474** | **1.016881** | **2.78^-69^** | **3.93^-69^** | **1.53434** | **1.252781** | **1.60128** | **0.37847** |
|  |  | Contactin-associated protein 1 | **0.2088** | **0.035259** | **0.25684** | **0.023106** | **0.55191** | **0.316342** | **0.83552** | **0.223943** |

**Table S6**: Motif analysis of putative anuran taxonomically-restricted learning-related genes. All differentially expressed genes showing only anuran orthologs were subjected to a protein motif search. The search produced 23 of 39 orthologs containing a protein motif. *Prosite score* represents the quality of the sequence match, a score above 6.50 is generally considered a strong match (green), while a score below 6.50 is a weak match (red). *ORF* represents the open reading frame within which the motif was found. *Treatment* represents gene expression log_2_ fold-change at 2, 4, and 24 hours post-training compared to control.

|  | Treatment | | |  |  |  |  |  |
| --- | --- | --- | --- | --- | --- | --- | --- | --- |
| Transcript I.D. | 2 Hours | 4 Hours | 24 Hours | Contig Length | Motif | Prosite score | ORF | Ascension |
| TRINITY_DN129288_c5_g2_i8 | -10.97 |  |  | 1172 | \ |  |  |  |
| TRINITY_DN130290_c1_g1_i3 | -10.67 |  |  | 2173 | Phenylalanine-rich region | 14.70 | -2 | PS50314 |
| TRINITY_DN132266_c0_g1_i2 | -10 |  |  | 703 | \ |  |  |  |
| TRINITY_DN133453_c5_g1_i5 | -8.22 |  |  | 1842 | NHL repeat | 4.46 | 1 | PS51125 |
| TRINITY_DN134765_c3_g1_i1 | -8.1 |  |  | 1718 | Uncharacterized conserved protein | 10.56 | 3 | DUF2373 |
| TRINITY_DN140713_c7_g1_i15 | -3.14 |  |  | 880 | \ |  |  |  |
| TRINITY_DN141337_c4_g6_i1 | 2.2 |  |  | 649 | Bipartite nuclear localization signal | 5.00 | -2 | PS50079 |
| TRINITY_DN143598_c4_g1_i1 | 2.69 |  |  | 248 | \ |  |  |  |
| TRINITY_DN145711_c11_g1_i2 | 2.71 | 2.54 |  | 270 | \ |  |  |  |
| TRINITY_DN146095_c0_g2_i7 | 3.47 |  |  | 1286 | Phenylalanine-rich region | 7.88 | 2 | PS50314 |
| TRINITY_DN146095_c0_g2_i9 | 6.12 |  |  | 1741 | BRCA2 repeat | 7.29 | -2 | PF00634 |
| TRINITY_DN148167_c1_g1_i5 | 7.06 |  | 7.76 | 1695 | Uncharacterized conserved protein | 9.82 | 1 | DUF2373 |
| TRINITY_DN150649_c11_g1_i1 |  | -8.23 |  | 800 | DNA/RNA helicase domain (DEAD box) | 6.60 | 2 | PS50136 |
| TRINITY_DN150735_c0_g2_i3 |  | -8.06 |  | 1365 | Lysine-rich region | 9.36 | 3 | PS50318 |
| TRINITY_DN150876_c3_g1_i1 |  | -2.42 |  | 1407 | Big-1 (bacterial Ig-like domain 1) | 4.69 | -3 | PF02369 |
| TRINITY_DN150876_c3_g1_i2 |  | -1.85 |  | 383 | Glutamine-rich region | 15.69 | -2 | PS50322 |
| TRINITY_DN151273_c2_g1_i4 |  | -1.63 |  | 973 | \ |  |  |  |
| TRINITY_DN153426_c5_g1_i2 |  | 1.82 |  | 515 | Bipartite nuclear localization signal | 4.00 | 1 | PS50079 |
| TRINITY_DN153573_c8_g1_i6 |  | 2.03 | 1.82 | 267 | Lysine-rich region | 10.36 | 2 | PS50318 |
| TRINITY_DN153983_c5_g1_i4 |  | 3.43 |  | 1769 | Serine-rich region | 6.63 | 2 | PS50324 |
| TRINITY_DN154332_c7_g1_i1 |  | 4.35 | 4.8 | 3035 | \ |  |  |  |
| TRINITY_DN155146_c2_g2_i5 |  | 4.82 |  | 3149 | \ |  |  |  |
| TRINITY_DN155167_c3_g2_i2 |  | 8.58 | 8.27 | 965 | Lysine-rich region | 12.23 | -3 | PS50318 |
| TRINITY_DN157130_c6_g1_i7 |  | 12.08 |  | 1664 |  |  |  |  |
| TRINITY_DN167692_c3_g2_i1 |  |  | -8.57 | 1379 | \ |  |  |  |
| TRINITY_DN164828_c0_g1_i4 |  |  | -8.18 | 359 | Protein of unknown function | 10.34 | -1 | DUF820 |
| TRINITY_DN163991_c4_g2_i5 |  |  | -6.98 | 1879 | Phenylalanine-rich region | 9.09 | -2 | PS50314 |
| TRINITY_DN159616_c3_g1_i17 |  |  | -6.8 | 2321 | Phenylalanine-rich region | 12.40 | -3 | PS50314 |
| TRINITY_DN166305_c8_g1_i3 |  |  | -4.08 | 1340 | Bipartite nuclear localization signal | 3.00 | 3 | PS50079 |
| TRINITY_DN167929_c13_g1_i2 |  |  | -3.42 | 577 | Bipartite nuclear localization signal | 3.00 | 1 | PS50079 |
| TRINITY_DN158727_c0_g2_i2 |  |  | -3.16 | 1628 | FtsK domain | 4.94 | 2 | PS50901 |
| TRINITY_DN165752_c2_g3_i2 |  |  | -2.66 | 2227 | \ |  |  |  |
| TRINITY_DN157436_c3_g1_i12 |  |  | -1.81 | 503 | Lysine-rich region | 8.35 | -1 | PS50318 |
| TRINITY_DN159758_c5_g2_i2 |  |  | -1.46 | 496 | \ |  |  |  |
| TRINITY_DN164242_c5_g1_i3 |  |  | -1.27 | 1188 | \ |  |  |  |
| TRINITY_DN161895_c3_g1_i1 |  |  | 2.3 | 1197 | ADF-H domain | 5.03 | 1 | PS51263 |
| TRINITY_DN162399_c1_g1_i11 |  |  | 5.83 | 2570 | \ |  |  |  |
| TRINITY_DN160910_c4_g1_i2 |  |  | 5.95 | 304 | \ |  |  |  |
| TRINITY_DN157586_c3_g1_i7 |  |  | 10.72 | 1129 | \ |  |  |  |

**Table S7:** Average transcripts per million (TPM) of the most relevant differentially expressed genes at 2h, 4h, and 24h post-conditioning. *IEG* groups represents any gene categorized as an immediate early gene. *General learning-related* group represents highly relevant mammalian learning-related genes that do not fall into other categories. *Structural* group represents any gene involved in cytoskeletal modification. *Guidance* group represents any gene involved in axon guidance. *Apoptotic* group represents any gene involved in programmed cell death. Gene selection was based on GO functional group enrichment analysis cross-referenced against the mammalian learning literature. Average TPM values for transcripts are reported. TPM values were obtained from the *eXpress* read alignment tool and were averaged across replicates (+/- StErr).

|  |  | Average TPM's (+/- StErr) | | | | | | |  |  |
| --- | --- | --- | --- | --- | --- | --- | --- | --- | --- | --- |
| Group | Gene | Control | +/- StErr | 2 Hours | +/- StErr | 4 Hours | +/- StErr | 24 Hours | | +/- StErr |
| IEG | Proto-oncogene c-Fos *(c-fos)* | **0.065** | **0.012** | **2.557** | **1.737** | **0.294** | **0.099** | **0.147** | | **0.054** |
|  | FosB Proto-Oncogene *(fosb)* | **0.002** | **0.001** | **0.108** | **0.051** | **0.249** | **0.048** | **0.039** | | **0.010** |
|  | Activity Regulated Cytoskeleton Associated Protein *(arc/arg1.3)* | **4.891** | **0.215** | **20.868** | **1.412** | **15.307** | **1.768** | **8.780** | | **1.307** |
|  | Nuclear Receptor Subfamily 4 Group A Member 1 *(Nr4a1)* | **1.964** | **0.498** | **10.740** | **0.500** | **6.408** | **0.579** | **4.876** | | **1.014** |
|  | Nuclear Receptor Subfamily 4 Group A Member 3 *(Nr4a3)* | **0.655** | **0.155** | **1.132** | **0.189** | **3.047** | **0.413** | **1.263** | | **0.246** |
|  | JunD Proto-Oncogene *(Jund)* | **10.573** | **0.574** | **22.051** | **1.833** | **21.220** | **1.342** | **15.664** | | **0.580** |
|  | Early Growth Response 1 *(egr1)* | **0.512** | **0.095** | **1.988** | **0.212** | **0.560** | **0.058** | **0.487** | | **0.204** |
|  | Immediate Early Response 2 *(ier2/pip92)* | **6.259** | **0.449** | **15.182** | **1.312** | **8.701** | **0.345** | **7.313** | | **0.259** |
|  | Neuronal PAS domain containing protein 4B *(Nps4b)* | **0.667** | **0.040** | **2.677** | **0.316** | **1.341** | **0.098** | **1.125** | | **0.025** |
|  | Homer Scaffold Protein 1 *(homer1)* | **0.119** | **0.021** | **0.063** | **0.018** | **0.028** | **0.013** | **0.089** | | **0.030** |
|  | Solute Carrier Family 2 Member 3 *(lc2a3)* | **0.244** | **0.139** | **0.122** | **0.051** | **0.468** | **0.190** | **0.229** | | **0.112** |
| General learning-related | Calcium/Calmodulin Dependent Protein Kinase II Alpha *(camk2a)* | **0.627** | **0.207** | **0.774** | **0.123** | **1.123** | **0.429** | **1.353** | | **0.160** |
|  | Brain Derived Neurotrophic Factor *(bdnf)* | **0.091** | **0.032** | **1.199** | **0.520** | **2.643** | **1.169** | **3.133** | | **0.569** |
|  | Neuronal Calcium Sensor 1 *(ncs-1)* | **0.482** | **0.196** | **0.370** | **0.041** | **0.451** | **0.171** | **0.001** | | **0.001** |
|  | Neuron Derived Neurotrophic Factor *(ndnf)* | **0.511** | **0.168** | **1.944** | **0.965** | **0.077** | **0.062** | **0.403** | | **0.152** |
|  | Solute Carrier Family 16 Member 1 *(slc16a1)* | **0.340** | **0.040** | **0.381** | **0.108** | **0.426** | **0.045** | **0.424** | | **0.010** |
|  | Nerve Growth Factor *(ngf)* | **100.578** | **7.788** | **114.626** | **7.901** | **98.544** | **8.557** | **84.443** | | **5.771** |
|  | Gamma-Aminobutyric Acid Type B Receptor Subunit 1 *(gabbr1)* | **6.624** | **0.376** | **7.649** | **0.908** | **7.580** | **0.032** | **8.433** | | **0.960** |
|  | Glutamate Metabotropic Receptor 5 *(grm5)* | **10.486** | **0.897** | **22.548** | **1.520** | **13.268** | **1.471** | **11.110** | | **2.874** |
| Structural | Glutamate Ionotropic Receptor AMPA Type Subunit 1 *(gria1)* | **0.378** | **0.102** | **5.222** | **2.217** | **1.735** | **1.212** | **2.857** | | **2.149** |
|  | Tropomodulin 2 *(tmod2)* | **0.076** | **0.035** | **2.707** | **1.271** | **0.304** | **0.221** | **1.364** | | **1.093** |
|  | Tropomodulin 3 *(tmod3)* | **0.007** | **0.006** | **0.238** | **0.016** | **0.078** | **0.064** | **0.329** | | **0.194** |
|  | IQ Motif And Sec7 Domain 3 *(Iqsec3)* | **21.721** | **0.578** | **47.810** | **8.923** | **28.566** | **4.435** | **27.370** | | **0.769** |
|  | L1 Cell Adhesion Molecule (*l1cam*) | **0.769** | **0.303** | **0.018** | **0.015** | **0.150** | **0.122** | **0.589** | | **0.379** |
|  | Unc-51 Like Autophagy Activating Kinase 1 *(ulk1)* | **113.843** | **14.923** | **47.532** | **2.877** | **81.521** | **20.719** | **38.329** | | **12.347** |
|  | GABA Type A Receptor-Associated Protein *(gabarap)* | **2.087** | **0.694** | **1.952** | **0.833** | **0.058** | **0.025** | **2.720** | | **0.349** |
|  | Catenin Alpha 2 *(ctnna2)* | **5.133** | **0.132** | **1.571** | **0.124** | **3.322** | **0.572** | **5.287** | | **0.615** |
|  | Catenin Beta 2 *(ctnnb2)* | **2.824** | **0.996** | **5.764** | **2.619** | **0.142** | **0.061** | **5.893** | | **4.640** |
|  | Synaptosome Associated Protein 91 *(snap91)* | **0.002** | **0.001** | **0.482** | **0.240** | **0.787** | **0.322** | **0.848** | | **0.063** |
|  | FRY Microtubule Binding Protein *(fry)* | **0.047** | **0.002** | **0.208** | **0.019** | **0.131** | **0.008** | **0.104** | | **0.008** |
|  | FRY Like Transcription Coactivator *(fryl)* | **0.292** | **0.031** | **0.706** | **0.232** | **0.286** | **0.106** | **0.898** | | **0.102** |
|  | BAI1 Associated Protein 2 *(baiap2)* | **0.025** | **0.013** | **0.178** | **0.145** | **0.968** | **0.375** | **0.394** | | **0.161** |
| Guidance | Semaphorin 3D *(sema3d)* | **2.256** | **0.576** | **3.962** | **0.808** | **2.399** | **0.891** | **8.059** | | **1.658** |
|  | Semaphorin 6A *(sema6a)* | **0.731** | **0.194** | **0.212** | **0.173** | **0.018** | **0.014** | **0.211** | | **0.087** |
|  | Semaphorin 7A *(sema7a)* | **0.000** | **0.000** | **0.000** | **0.000** | **0.000** | **0.000** | **7.022** | | **3.047** |
|  | EPH Receptor A3 *(epha3)* | **0.000** | **0.000** | **0.889** | **0.200** | **0.560** | **0.244** | **0.606** | | **0.309** |
|  | EPH Receptor B2 *(ephb2)* | **0.286** | **0.025** | **0.097** | **0.002** | **0.169** | **0.034** | **0.209** | | **0.051** |
|  | EPH Receptor 8A *(epha8)* | **1.229** | **0.158** | **1.392** | **0.033** | **2.859** | **0.271** | **1.188** | | **0.051** |
|  | Ephrin A5 *(efna5)* | **0.764** | **0.199** | **4.186** | **1.903** | **2.603** | **1.749** | **0.123** | | **0.043** |
|  | Neuropilin 1 *(nrp-1)* | **0.986** | **0.094** | **3.213** | **2.379** | **4.995** | **1.671** | **2.651** | | **0.891** |
|  | Neuropilin 2 *(nrp-2)* | **0.553** | **0.094** | **0.747** | **0.233** | **0.737** | **0.230** | **1.535** | | **0.220** |
|  | Rho Guanine Nucleotide Exchange Factor 12 *(arhgef12)* | **0.313** | **0.023** | **0.025** | **0.009** | **0.103** | **0.068** | **0.125** | | **0.025** |
|  | Neuroepithelial Cell Transforming 1 *(net1)* | **0.012** | **0.009** | **0.194** | **0.105** | **0.336** | **0.140** | **0.230** | | **0.028** |
|  | Slit Guidance Ligand 2 *(slit2)* | **1.150** | **0.281** | **0.009** | **0.007** | **0.755** | **0.307** | **0.680** | | **0.292** |
|  | Roundabout Guidance Receptor 2 *(robo2)* | **0.549** | **0.063** | **0.014** | **0.011** | **0.002** | **0.001** | **0.145** | | **0.119** |
| Apoptotic | Fas Cell Surface Death Receptor *(fas)* | **0.040** | **0.031** | **0.422** | **0.344** | **3.674** | **1.444** | **2.582** | | **1.041** |
|  | Fas Associated Factor 1 *(faf1)* | **1.026** | **0.515** | **0.553** | **0.320** | **0.431** | **0.119** | **0.033** | | **0.020** |
|  | Death Domain Associated Protein *(daxx)* | **0.095** | **0.039** | **0.363** | **0.014** | **0.122** | **0.068** | **0.777** | | **0.080** |
|  | Programmed Cell Death 4 *(pdcd4)* | **0.182** | **0.033** | **0.268** | **0.122** | **0.083** | **0.052** | **3.213** | | **2.225** |
|  | Protein Kinase C Delta *(prkcd)* | **2.431** | **0.621** | **5.939** | **1.589** | **8.007** | **2.657** | **7.161** | | **1.411** |
|  | Death Inducer-Obliterator 1 *(dido1)* | **3.131** | **1.981** | **2.956** | **0.443** | **5.138** | **1.239** | **0.000** | | **0.000** |
|  | Bifunctional Apoptosis Regulator *(bfar)* | **0.065** | **0.012** | **2.557** | **1.737** | **0.294** | **0.099** | **0.147** | | **0.054** |


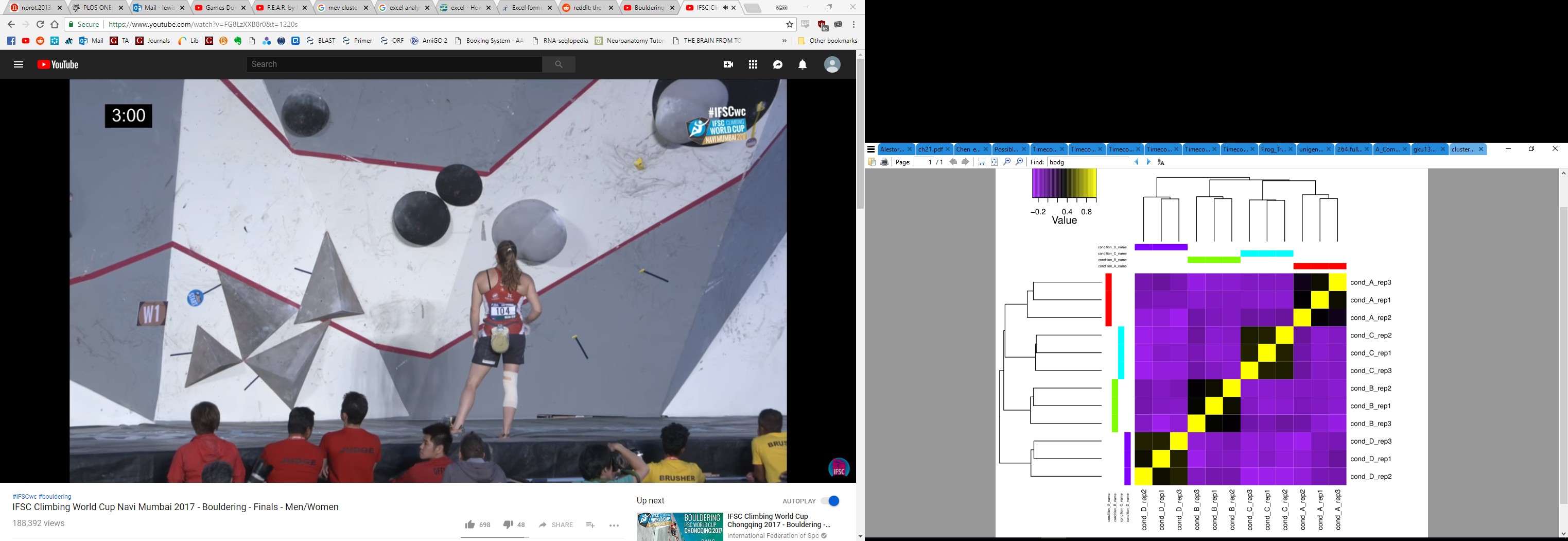


Condition A (Control):

Condition B (2 Hours):

Condition C (4 Hours):

Condition D (24 Hours):

**Figure S1:** Correlation matrix comparing biological replicate variability. The figure is based on variability in the expression data matrix produce by the DESeq2 differential expression analysis. Black/yellow cells indicate greater within replicate similarity than between replicate similarity.

**Figure S2**: Preliminary experiment on the effect of delay between training sessions on acquisition of the prey-catching response. Twelve fire-bellied toads were subjected to 12 prey-catching conditioning trials distributed over two sessions, with the sessions occurring either on the same day (between-session interval of 30 min; 2/d – black bars) or over two days (between-session interval of 24 h; 1/d – white bars). Data are mean + SEM. An equivalent decrease in the time to reach five snaps at the cricket stimulus was found in both groups (session effect: F(1,9)=20.54, p = 0.0014; group effect and session*group interaction: p > 0.05).

**Figure S3:** Individual prey-catching conditioning response curves of the 12 toads used for genomic analysis. The toads were subjected to 12 conditioning trials across two sessions separated by an interval of 30 min. Inter-trial interval within sessions was 3 minutes. Brain tissue was collected at 2 h, 4 h, or 24 h after conditioning to assess temporal profiles of gene expression.

*Section 2: RNA quality and extraction protocol*

**Figure S4:** Nanodrop results of RNA extraction for all samples used in this study.


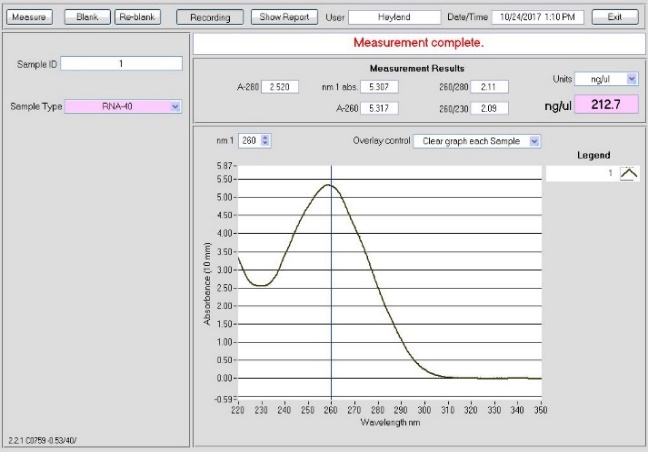

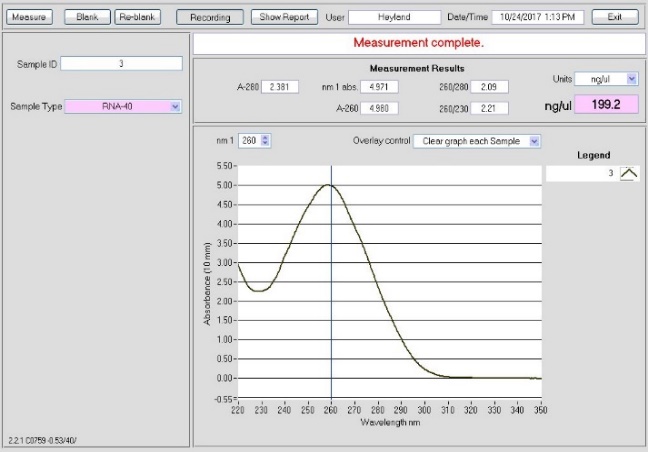

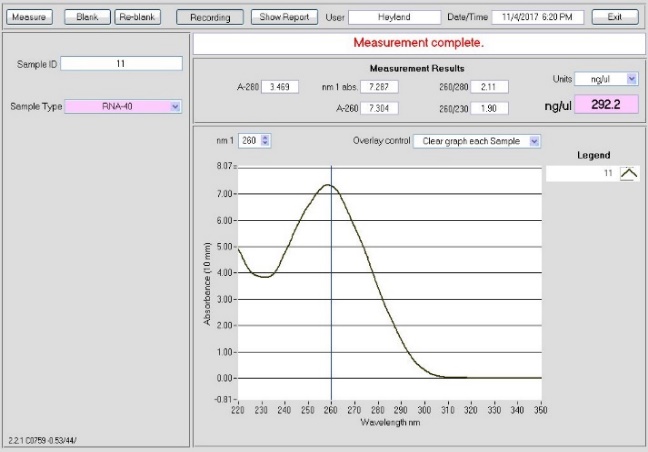

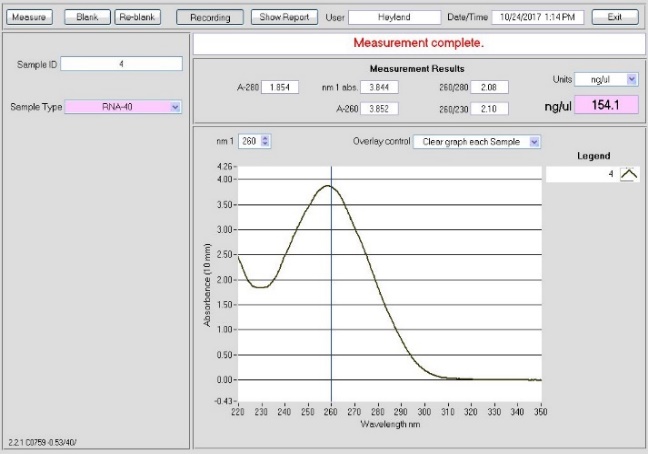

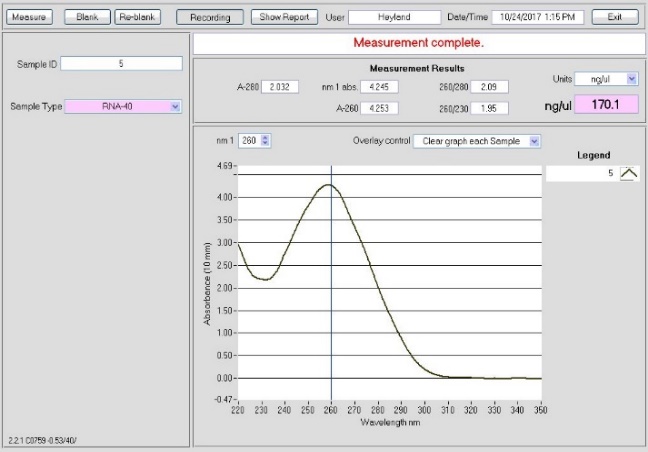

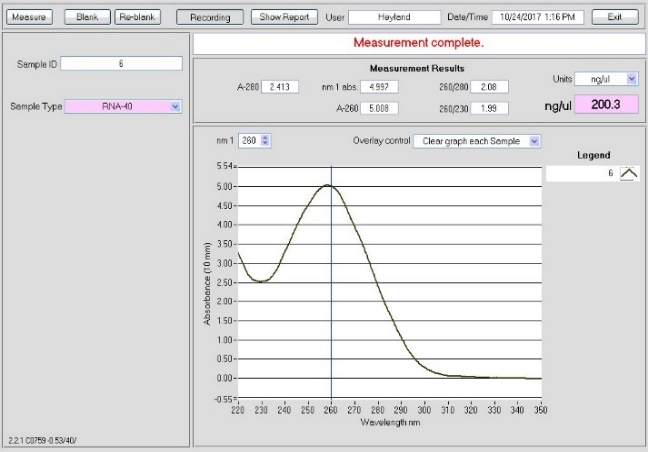

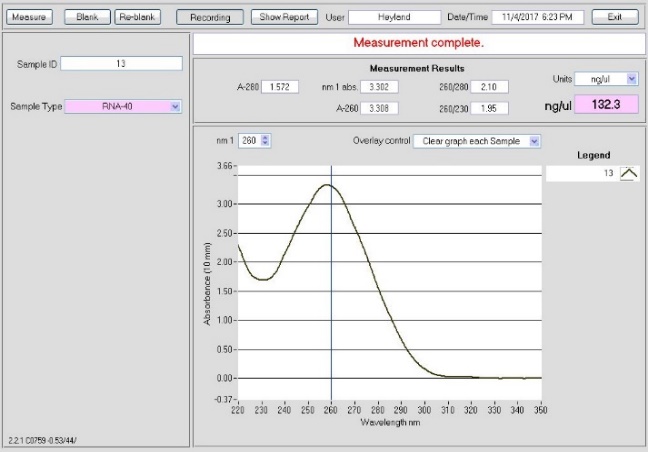

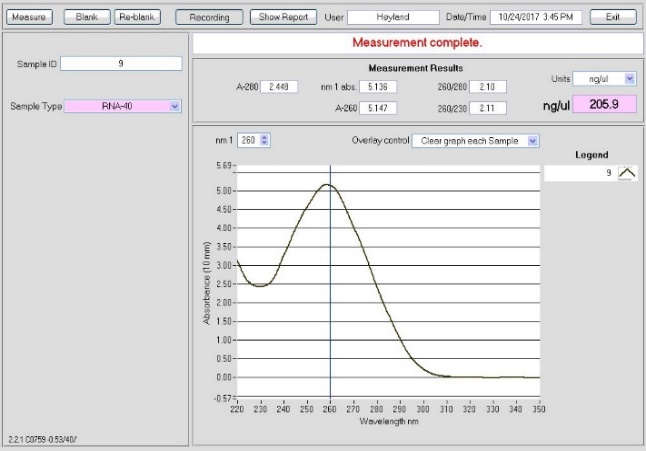

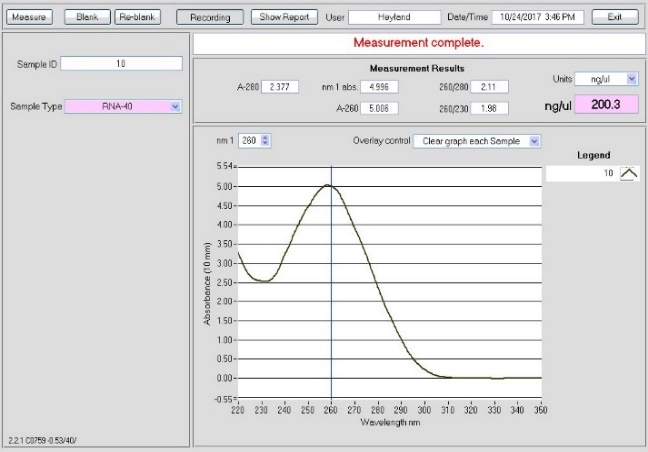

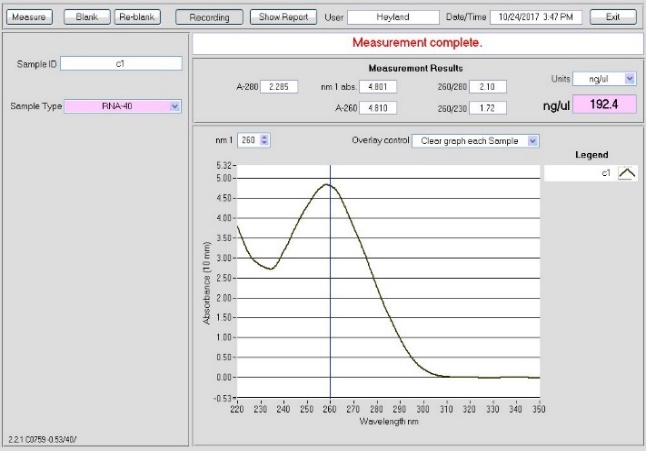

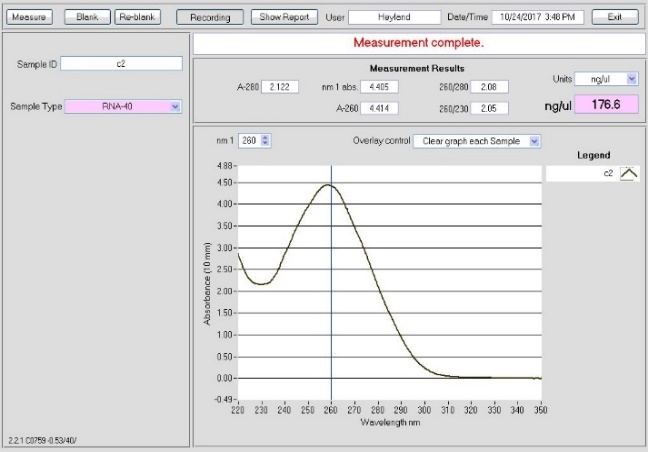

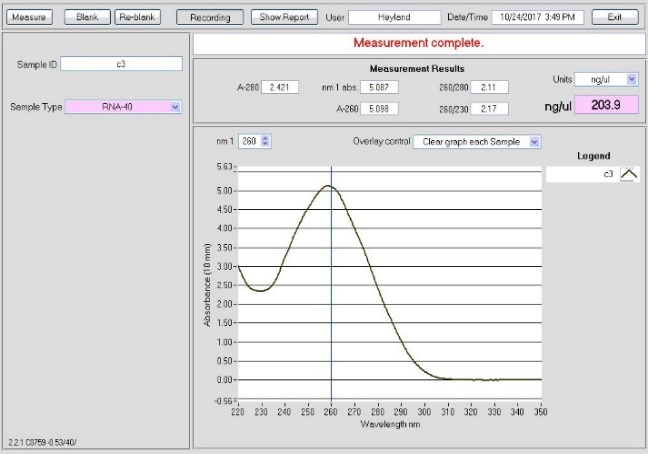


Control

2 Hours

4 Hours

24 Hours

Replicate 1

Replicate 2

Replicate 3

**Table S8:** RNA integrity analysis for all samples used in this study. Analysis was done by The Hospital for Sick Children’s Center for Applied Genomics (TCAG, Toronto, ON).

| Sample | R.I.N. | RNA concentration ng/ul | 28s/18s ratio |
| --- | --- | --- | --- |
| Control -1 | 9 | 156 | 1.1 |
| Control -2 | 9.3 | 157 | 1.1 |
| Control -3 | 9 | 160 | 1.1 |
| 2 hours -1 | 9.5 | 130 | 1.3 |
| 2 hours -2 | 9.5 | 298 | 1.2 |
| 2 hours -3 | 8.7 | 137 | 1.2 |
| 4 hours -1 | 9.3 | 177 | 1.2 |
| 4 hours -2 | 9.7 | 113 | 1.2 |
| 4 hours -3 | 9.6 | 154 | 1.2 |
| 24 hours -1 | 9.5 | 108 | 1.4 |
| 24 hours -2 | 9.5 | 229 | 1.2 |
| 24 hours -3 | 9.2 | 198 | 1.1 |

**RNA extraction protocol**

1. Tissue extraction: Brain removed in fresh prepared, cooled ringer solution (Medulla –olfactory bulb).
2. Remove fresh tissue to 1ml TRIzol™ in 1.5 Eppendorf tube on dry ice.
3. RNA Extraction: Thaw tissue, spin down ~10seconds, grind tissue with pestle.
4. Centrifuge for 10 min, at 12000g/4C. Transfer supernatant to new tube, discard debris pellet.
5. Incubate 5min, at room temp.
6. Add 250ul Chloroform and shake ~1min
7. Incubate 15min at room temp
8. Centrifuge for 15min, at 12000g/4C. Transfer aqueous phase, discard rest.
9. Add 500ul isopropyl alcohol, 100ul sodium acetate, 1ul glycogen. Vortex quickly.
10. Incubate -20C (24h).
11. Centrifuge for 10min, at 12000g/4C. Remove supernatant, keep pellet.
12. Add 1 volume 75% ethyl alcohol (EtOH), shake ~5sec.
13. Centrifuge for 5min, at 7500g/4C. Decant EtOH. Repeat once.
14. Following final EtOH wash, decant and air-dry pellet for ~5min.
15. Elute in 100ul Nuclease-free-water.
16. RNA clean-up (RNeasy mini kit: Qiagen, Venlo, Netherlands): Add 350ul RLT buffer and mix.
17. Add 250ul EtOH, mix with pipette.
18. Transfer to spin column, spin for 15sec at 8000g. Discard flow through.
19. DNase column digestion steps:
    1. Add 350ul RW1 buffer, spin 15sec at 8000g. Discard flow through.
    2. Mix 10ul DNAse 1 and 70ul RDD buffer, spin quick.
    3. Add mix to column incubate at room temp for 15min
    4. Add 350ul RW1 buffer to column, spin 15sec at 8000g.
20. Add 500 RPE buffer to column, spin 15sec at 8000g.
21. Place column in 1.5 ml tube, add 50ul Nuclease-free-water and spin for 1min at 8000g.
22. Nanondrop and store (-20C)
